# A novel 4-aminoantipyrine-Pd(II) complex catalyzes Suzuki–Miyaura cross-coupling reactions of aryl halides

**DOI:** 10.3762/bjoc.10.299

**Published:** 2014-12-01

**Authors:** Claudia Araceli Contreras-Celedón, Darío Mendoza-Rayo, José A Rincón-Medina, Luis Chacón-García

**Affiliations:** 1 Laboratorio de Síntesis y Diseño Molecular, Instituto de Investigaciones Químico Biológicas, Universidad Michoacana de San Nicolás de Hidalgo. Edificio B-1, Ciudad Universitaria, Morelia, Michoacán, México. CP 58030, tel.: +52 443 326 5790; fax: +52 443 326 5788

**Keywords:** 4-aminoantipyrine, arylboronic acids, biaryls, cross-coupling, palladium(II) complex

## Abstract

A simple and efficient catalytic system based on a Pd complex of 4-aminoantipyrine, 4-AAP–Pd(II), was found to be highly active for Suzuki–Miyaura cross-coupling of aryl iodides and bromides with phenylboronic acids under mild reaction conditions. Good to excellent product yields from the cross-coupling reaction can be achieved when the reaction is carried out in ethanol, in the open air, using low loading of 4-AAP–Pd(II) as a precatalyst, and in the presence of aqueous K_2_CO_3_ as the base. A variety of functional groups are tolerated.

## Introduction

The sp^2^–sp^2^ carbon–carbon bond formation through cross-coupling reactions catalyzed by metal complexes has emerged as a powerful tool in organic synthesis [1–6]. The palladium-catalyzed cross-coupling of arylboronic acid and aryl halides in the Suzuki–Miyaura (SM) reaction is one of the most popular and important methods to obtain biaryls, which are essential structures of many important compounds such as natural products [7], agrochemicals [8], pharmaceuticals [9] and polymers [10] among others. The broad application of the SM coupling arises from the exceptionally mild reaction conditions, the tolerance to different functional groups, the relatively stable, readily prepared and generally environmentally benign nature of the oroganoboron reagents, and their rapid transmetalation with palladium(II) complexes [11].

Although in recent years there have been numerous studies on the SM cross-coupling reaction, the necessity for a simple procedure that allows the formation of C–C bonds in functionalized substrates remains. There have been ongoing efforts to develop a stable and efficient Pd catalyst for these reactions. Recently, palladium complexes containing imidazole-imines [12], binary nanoclusters [13], N-heterocyclic carbenes (NHCs) [14], nanoparticles [15], palladacycles [16], and Schiff bases [17] have been developed as highly effective phosphine-free catalysts for SM coupling reactions.

Transition metal complexes that have shown a wide range of biological activity are those containing the pyrazolone derivative 4-aminoantipyrine (4-amino-1,5-dimethyl-2-phenyl-1*H*-pyrazol-3(2*H*)-one, or simply “4-AAP”) [18]. Pyrazoles, in general, are one of the most important classes of bioactive heterocycles, having attracted increasing interest to the pharmaceutical, chemical and agricultural industries over the past decade, and in recent years a number of research articles have been published specifically about 4-AAP [19]. Analogues and transition metal complexes of 4-AAP have shown anti-inflammatory, analgesic, antiviral, antipyretic, antirheumatic and antimicrobial activity [20–21]. Despite the potential biological importance of 4-AAP, the catalytic activity of its transition metal complexes for C–C bond formation have not yet been investigated. Herein, we report the synthesis of the new 4-aminoantipyrine–Pd(II) complex [4-AAP–Pd(II)] by mixing Li_2_PdCl_4_, 4-aminoantipyrine in presence of NaOAc in MeOH at room temperature, and its performance as catalysts in SM cross-coupling reaction of structurally different aryl halides with phenylboronic acids. The biaryls are obtained in moderate to high yield.

## Results and Discussion

**Scheme 1:**
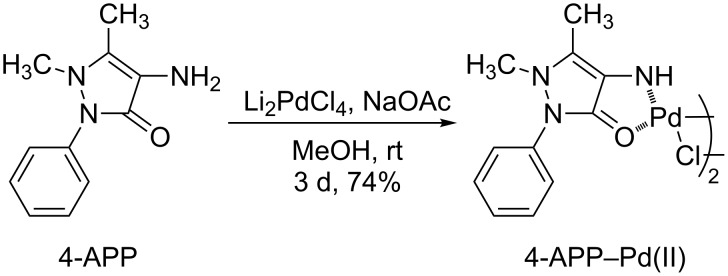
Synthesis of 4-aminoantipyrine-Pd(II) complex.

The synthesis of the 4-APP–Pd(II) complex was carried out using a convenient one-pot procedure, as reported in the literature for other ligands [22], by combining Li_2_PdCl_4_, 4-AAP and NaOAc in methanol at room temperature (Scheme 1).

The 4-AAP–Pd(II) complex is thermally stable, not sensitive to oxygen or moisture, and highly soluble in common solvents such as CHCl_3_, CH_2_Cl_2_ and AcOEt. The newly synthesized 4-AAP–Pd(II) complex was characterized by UV–vis, FTIR, ^1^H, ^13^C NMR spectroscopy and MS (Table 1). The UV–vis spectra in CH_3_CN showed a broad band at approximately 465 nm. In the IR spectra, the characteristic NH and C=O stretching absorption bands appeared around 3476 and 1616 cm^−1^, respectively. Anal. calcd for C_22_H_24_Cl_2_N_6_O_2_Pd_2_ C 38.39, H 3.51, N 12.21; found: C 38.52, H 3.54, N 12.35. The ^1^H NMR spectra showed signals at 7.55–7.44 and 7.33–7.42 ppm, integrating for five aromatics protons. The signals for the N-CH_3_ and the C-CH_3_ protons were shifted upfield from their positions in uncomplexed 4-AAP, being observed as singlets at 3.07 and 2.29 ppm respectively. The NH signal was similarly shifted upfield, to 1.84 ppm, with its integration indicating one proton. This result suggests to us that the palladium interacts with the carbonyl and amine groups of the pyrazole ring of 4-AAP.

**Table 1:** Spectroscopic data for 4-AAP and 4-APP–Pd(II) complex.

	Compound	Signals

IR	4-AAP	3432, 3325 (NH_2_), 3246 (N-CH_3_), 2914 (CH-Ar), 1643 (C=O)
	4-APP–Pd(II)	3474 (NH), 2924 (CH-Ar), 1616 (C=O)
^1^H NMR (CDCl_3_)	4-AAP	7.5–7.4 (2H-Ar), 7.3–7.2 (3H-Ar), 2.95 (2H, NH_2_), 2.84 (3H, N-CH_3_), 2.15 (3H, C-CH_3_)
	4-APP–Pd(II)	7.55–7.44 (2H-Ar), 7.42–7.33 (3H-Ar), 1.84 (1H, NH), 3.07 (3H, N-CH_3_), 2.29 (3H, C-CH_3_)
^13^C NMR (CDCl_3_)	4-AAP	162.0 (C=O), 138.0, 118.9 (N-C=C), 135.3, 128.9, 125.8, 122.7 (C-Ar), 37.8 (N-CH_3_), 10.1 (C-CH_3_)
	4-APP–Pd(II)	161.4 (C=O), 133.2, 112.3 (N-C=C), 143.7, 129.3, 129.0, 127.8, 124.7, 123.3 (C-Ar), 35.4 (N-CH_3_), 10.3 (C-CH_3_)

The synthesized 4-AAP–Pd(II) complex was used to carry out all palladium-catalyzed Suzuki–Miyaura cross-coupling reactions described in this report. We screened various solvents, bases, and percents of precatalyst loaded, in order to optimize the reactions conditions, and then applied these optimized conditions to reactions on a wide scope of substrates. For each of the three screening experiments, phenylboronic acid (**1a**) and *p*-bromobenzaldehyde (**2e**) were chosen as the model substrates, which in the coupling reaction produced the biphenyl **3q** (see Table 2 and Table 3). We first describe the results of these screens. The screen for solvents was carried out using 0.3 mol % of the palladium complex as a catalyst and many bases; the results are summarized in Table 2. The reaction proceeded in both protic and aprotic solvents, but with significantly varying yields of the product. In the cases of H_2_O, THF, DMF, toluene, benzene and PEG 600 solvents, the yields were between 29–67% (Table 2, entries 1–6), all lower than the product yield obtained when using methanol or ethanol as the solvent (Table 2, entries 8–10). In the case of ethanol (Table 2, entries 9 and 10) the percent yield of product **3q** was relatively high (89%) when the reaction was carried out with heating under reflux for 4 hours, but was also similarly high when the reaction was carried out at room temperature for 22 hours. However, the yield of **3q** was low when 1:1.5 PEG 600/H_2_O was used at 80 °C (Table 2, entry 7) and moderate yield when 1:1 EtOH/H_2_O when was used as solvent at room temperature (rt) or at 80 °C (Table 2, entries 11 and 12). Different bases were screened using 0.3 mol % of the 4-AAP–Pd(II) complex in ethanol at reflux (Table 2). When the reaction (Table 2, entry 13) was conducted without any base, no reaction was observed even after 24 h at room temperature or reflux. In the case of NaOH (Table 2, entry 14) we observed a poor product yield (17%). In the presence of bases, such as NaOAc and K_3_PO_4_, moderate yields are obtained (Table 2, entries 15 and 16). Na_2_CO_3_ and K_2_CO_3_ were found to give the best yields (Table 2, entries 9 and 17). In entry 18, the reaction was carried out in the presence of Li_2_PdCl_4_ as catalyst and no reaction was observed even after 24 h at room temperature or reflux. The results shown in Table 2 indicate that aqueous K_2_CO_3_ or Na_2_CO_3_ as base and ethanol as solvent are the best conditions of those tested for the 4-AAP–Pd(II)-catalyzed cross-coupling reaction of phenylboronic acid (**1a**) with *p*-bromobenzaldehyde (**2e**).

**Table 2:** Screening of solvent and base for the 4-AAP-Pd(II)-catalyzed cross-coupling reaction.^a^

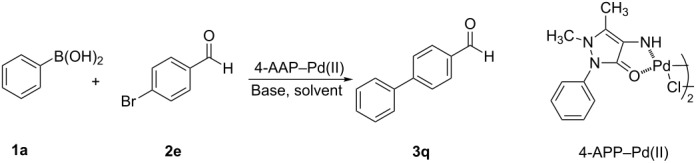				

Entry	Solvent	Base	Time (h)	**3q** yield (%)^b^

1	H_2_O	Na_2_CO_3_	4	47
2	THF	Na_2_CO_3_	8	29
3	DMF^c^	Na_2_CO_3_	3	37
4	toluene^c^	Na_2_CO_3_	3	36
5	benzene	Na_2_CO_3_	8	67
6	PEG 600^c^	Na_2_CO_3_	4	30
7	PEG 600/H_2_O (1:1.5)^c^	Na_2_CO_3_	4	33
8	MeOH	Na_2_CO_3_	3	75
9	EtOH	Na_2_CO_3_	4	89
10	EtOH^d^	Na_2_CO_3_	22	88
11	EtOH/H_2_O (1:1)	Na_2_CO_3_	3	69
12	EtOH/H_2_O (1:1)	Na_2_CO_3_	22	57
13	EtOH^a,d^	–	24	–
14	EtOH	NaOH	4	17
15	EtOH	NaOAC	4	59
16	EtOH	K_3_PO_4_	4	68
17	EtOH	K_2_CO_3_	4	89
18	EtOH^a,c,e^	K_2_CO_3_	24	–

^a^Reaction conditions: phenylboronic acid (**1a**, 0.40 mmol), *p*-bromobenzaldehyde (**2e**, 0.27 mmol), 4-AAP–Pd(II) (0.3 mol %), 2 M base (0.67 mmol) and solvent (2 mL) at reflux. ^b^Isolated yield. ^c^The reaction was carried out at 80 °C. ^d^The reaction was carried out at room temperature. ^e^The reaction was carried out in the presence of Li_2_PdCl_4_ as catalyst.

For the final screen, catalyst loading tests, at various relatively low levels of 4-AAP–Pd(II), were performed to determine its catalytic efficiency. This screen was carried out in the presence of aqueous K_2_CO_3_ as base and ethanol as solvent. Decreasing the concentration of catalyst from 0.3 mol % to 0.03 mol % substantially reduced the product yield (to 46%, see Table 3 entry 1) when the reaction was carried out under reflux for 4 h, but did not reduce the yield when the reaction was carried out at room temperature for 22 h (Table 3, entry 2). However, when using only 0.3 mol % of 4-AAP–Pd(II), the reaction proceeded with relatively high yield of product (88–89%) when carried out for up to 4 h with heating at reflux or at room temperature for 22 h (Table 3, entries 3 and 4). Increasing the concentration of catalyst, from 0.3 mol % to 3 mol % somewhat reduced the product yield, to moderate levels, when the reaction was carried out at either room temperature for 22 h or under reflux for 4 h (Table 3, entries 5 and 6). The decrease in the coupling yield at high concentrations of Pd(II) catalyst is a common phenomenon in palladium chemistry due to the aggregation of Pd(0) occurs [3,23].

**Table 3:** Effect of low precatalyst loading.^a^

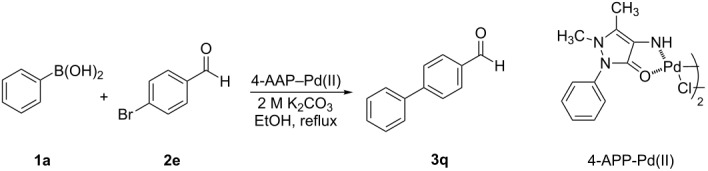				

Entry	precatalyst (mol % Pd)	Time (h)	**3q** Yield (%)^b^	TON^d^

1	0.03	4	46	3059
2	0.03^c^	22	88	5852
3	0.3	4	89	591
4	0.3^c^	22	88	585
5	3	4	82	55
6	3^c^	22	71	47

^a^Reaction conditions: phenylboronic acid (**1a**, 0.40 mmol), *p*-bromobenzaldehyde (**2e**, 0.27 mmol), 4-AAP-Pd(II), 2 M K_2_CO_3_, (0.067 mmol), ethanol (2 mL) heating under reflux. ^b^Isolated yield. ^c^The reaction was carried out at room temperature. ^d^TON = mol of product per mol of catalyst.

**Scheme 2:**
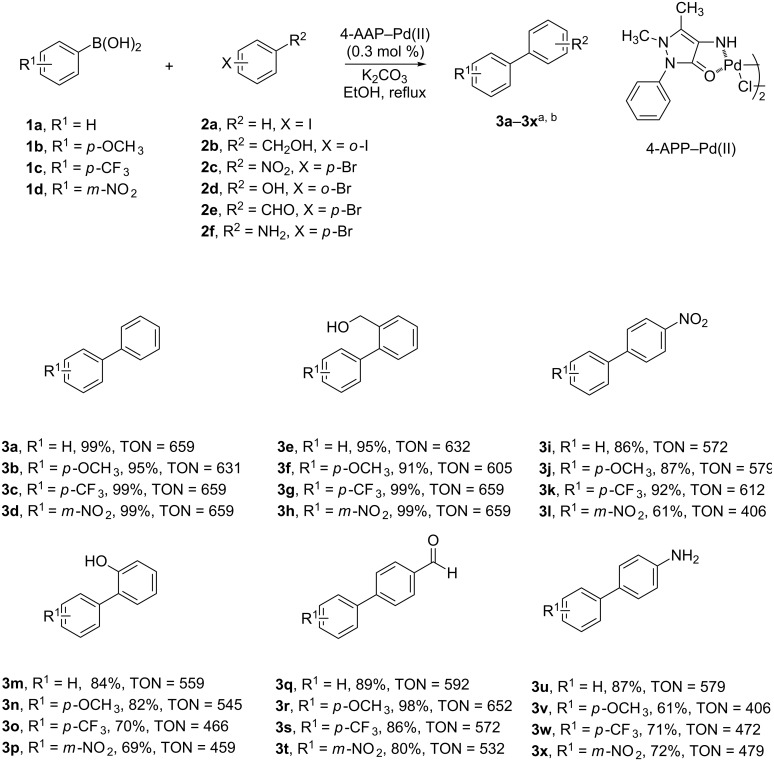
Reaction of different aryl halides with substituted arylboronic acids. Reaction conditions: phenylboronic acid (0.40 mmol), aryl halide (0.27 mmol), 4-AAP–Pd(II) (0.3 mol %), 2 M K_2_CO_3_ (0.67 mmol), ethanol (2 mL), heating under reflux for 4 h. ^a^Isolated yield. ^b^TON = mol of product per mol of catalyst.

In order to explore the scope of the SM cross-coupling reaction using the 4-AAP–Pd(II) complex, we examined the reactions of a wide array of electronically diverse aryl iodides and bromides with electron-withdrawing or electron-donating substituents on the phenylboronic acids. The effects of the substituents of the arylboronic acid on the product yield of this reaction have been analyzed. Of particular note is our observation that many products could be isolated without the need for column chromatography. Using this protocol, all 8 tested reactions of phenylboronic acids **1a**–**1d** with phenyl iodides **2a**, **2b** proceeded smoothly to produce the desired biphenyl compounds in high to excellent yields (91–99%) (Scheme 2, compounds **3a–3h**). The 16 reactions with aryl bromides **2c**–**2f** also proceeded well and afforded the corresponding biphenyl products (Scheme 2 compounds **3i–3x**) in moderate to high yields (61–98%). The catalytic system was tolerant for both electron-donating and electron-withdrawing substituents; our protocol successfully accommodated the free hydroxy and amino groups as substituents on the aryl iodides and bromides without additional protection procedures, and the reactions with these substrates produced the corresponding biphenyl compounds with moderate yields (Scheme 2 compounds **3e–3h**, **3m–3p**, **3u–3x)**. In addition to the yield, the turnover number (TON in Scheme 2) of the 4-AAP–Pd(II) catalyst was measured, and found to be moderate (400–700) for all 24 tested cross-coupling reactions, suggesting that the catalyst is also efficient.

## Conclusion

In summary, we have developed a mild, efficient and comparatively inexpensive methodology for the synthesis of biaryl compounds. This methodology uses our newly developed 4-AAP–Pd(II) complex as a highly efficient precatalyst and general catalyst for the SM cross-coupling, works without the necessity of phosphine ligands, and was also found to be active for the cross-coupling of aryl iodides and bromides with substituted phenylboronic acids. The SM cross-coupling reaction can be carried out in EtOH, in the presence of air, with low catalyst loadings, and heating at reflux conditions for relatively short reaction times to afford biaryl compounds in good to excellent yields. The synthetic accessibility and stability under cross-coupling reaction conditions of 4-AAP–Pd(II) make this complex a very promising precatalyst, and we will continue studying its applicability in various organic reactions.

## Supporting Information

File 1 Full experimental details and copies of all NMR spectra (^1^H and ^13^C spectra) of all compounds isolated.
