# Supplementary material for: A novel 4-aminoantipyrine-Pd(II) complex catalyzes Suzuki–Miyaura cross-coupling reactions of aryl halides
Source: Beilstein J Org Chem. 2014 Dec 1;10:2821–6. doi: 10.3762/bjoc.10.299 (PMC4273307; doi:10.3762/bjoc.10.299)
Supplement: File 1 — Full experimental details and copies of all NMR spectra (1H and 13C spectra) of all compounds isolated. [file Beilstein_J_Org_Chem-10-2821-s001.pdf]

Supporting Information

for

A novel 4-aminoantipyrine-Pd(II) complex catalyzes Suzuki–Miyaura cross-coupling reactions of aryl halides

Claudia A. Contreras-Celedón*, Darío Mendoza-Rayo, José A. Rincón-Medina and Luis Chacón-García

Address: Laboratorio de Síntesis y Diseño Molecular, Instituto de Investigaciones Químico Biológicas, Universidad Michoacana de San Nicolás de Hidalgo. Edificio B-1, Ciudad Universitaria, Morelia, Michoacán, México. CP 58030, tel.: +52 443 326 5790; fax: +52 443 326 5788

Email: Claudia A. Contreras Celedón - celedon@umich.mx

*Corresponding Author

Full experimental details and copies of all NMR spectra (^{1}H and ^{13}C spectra) of all compounds isolated

Table of Contents

General methods and materials S3

Synthesis and characterization of 4-aminoantipyrine palladium(II) complex, 4-AAP–Pd(II) S3

General procedure for the Suzuki–Miyaura cross-coupling reaction S4

Characterization of the products S4

^{1}H NMR and ^{13}C NMR spectra S12

References S39

General methods and materials: All reagents and solvents used were obtained commercially and used without further purification unless indicated otherwise. All products were characterized by ^{1}H NMR and ^{13}C NMR. ^{1}H NMR spectra were recorded on 400 MHz in $CDCl_3$, and ^{13}C NMR spectra were recorded on 101 MHz in $CDCl_3$ using TMS as internal standard. Multiplicities are indicated as s (singlet), d (doublet), t (triplet), q (quartet), and m (multiplet), and coupling constants (J) are reported in hertz.

Synthesis of 4-aminoantipyrine–palladium (II) complex, 4-AAP–Pd(II): [1] To a stirred solution 0.5 M of Li_2PdCl_4 (1.13 mL, 0.56 mmol) in methanol at room temperature was added a solution of 4-AAP (0.115 g, 0.56 mmol) in methanol (2mL) and AcONa (0.046 g, 0.56 mmol). Stirring was continued for 3 days and then the mixture was diluted with H_2O (2 mL). The precipitated compound was filtered, washed with H_2O (2 X 2 mL) and ethyl ether (2 x 2 mL). After recrystalization ($CHCl_3$:Hex) provided the 4-AAP-Pd(II) complex as orange solid. Yield 74%; mp 210°C; IR (neat): 3478, 2926, 1616, 1586 cm^{-1}; UV: λ^{max} 465 nm; ^{1}H-NMR (400 MHz, $CDCl_3$) δ ppm: 7.55 – 7.44 (m, 2H), 7.42 – 7.33 (m, 3H), 3.07 (s, 3H), 2.29 (s, 3H), 1.84 (q, 1H); ^{13}C-NMR ($CDCl_3$) δ ppm: 161.4, 143.7, 133.2, 129.3, 129.0, 127.8, 124,7, 123.3, 112.3, 35.4, 10.3; Anal. Calcd. for $C_{22}H_{24}Cl_2N_6O_2Pd_2$ C 38.39, H 3.51, N 12.21; found C 38.52, H 3.54, N 12.35.

General Procedure for the Suzuki–Miyaura cross-coupling reaction: In a 10 mL glass tube containing a Teflon-coated stir bar was placed p-bromobenzaldehyde **2e** (0.05 g, 0.27 mmol, 1 equiv), phenylboronic acid **1a** (0.05 g, 0.40 mmol, 1.5 equiv), 2M K_2CO_3 (0.33 mL, 0.67 mmol, 2.5 equiv), 4-AAP-Pd(II) (0.28 mg, 0.3 mol % Pd) and EtOH (2 mL). The mixture was stirred at reflux for 4 h. After cooling, the mixture was diluted with ether Et_2O (5 mL), washed with sat. aq. $NaHCO_3$ (3 mL), brine (3 mL) and dried over Na_2SO_4. Evaporation of the solvent and purification of the residue over a silica gel column (Hex: AcOEt 90:10), furnished the biphenyl **3q**.

Characterization of the products

1,1'-Biphenyl (**3a**): [2]

White solid; m.p. = 69-70°C; ^{1}H-NMR (400 MHz, $CDCl_3$): δ (ppm) 7.59 (dd, J = 8.2, 1.1Hz, 4H), 7.43 (m, 4H), 7.34 (m, 2H); ^{13}C-NMR (101 MHz, $CDCl_3$): δ (ppm) 141.2, 128.7, 127.2, 127.1

4-Methoxy-1,1'-biphenyl (**3b**): [3]

White solid, m.p. = 88-89°C; ^{1}H-NMR (400 MHz, $CDCl_3$): δ (ppm) 7.58-7.54 (m, 4H), 7.45-7.41 (m, 2H), 7.34-7.30 (m, 1H), 7.01-6.98 (m, 2H), 3.86 (m, 3H); ^{13}C-NMR (101 MHz, $CDCl_3$): δ (ppm) 159.9, 140.6, 133.6, 128.5, 128.0, 126.5, 126.5, 114.0, 55.2

4-(Trifluoromethyl)-1,1'-biphenyl (**3c**): [4]

White solid, m.p. = 66-67°C, ^{1}H-NMR (400 MHz, $CDCl_3$): δ (ppm) 7.69 (s, 4H), 7.60 (m, 2H), 7.48 (m, 2H), 7.41 (m, 1H); ^{13}C-NMR (101 MHz, $CDCl_3$): δ (ppm) 144.7, 139.7, 129.0, 128.2, 127.6, 127.4, 127.3, 125.73, 125.69, 125.65, 125.60

3-Nitro-1,1'-biphenyl (**3d**): [5]

Light yellow solid, m.p. 57-58°C, ^{1}H-NMR (400 MHz, $CDCl_3$): δ (ppm) 8.46 (t, J = 2Hz, 1H), 8.20 (ddd, J = 8.2, 2.3, 1.0Hz, 1H), 7.92 (dd, J=7.7, 1.8, 1.1Hz, 1H), 7.63 (m, 3H), 7.58 (m, 2H), 7.44 (m, 1H); ^{13}C-NMR (101 MHz, $CDCl_3$): δ (ppm) 148.5, 142.7, 138.5, 132.9, 129.5, 129.0, 128.4, 127.0, 121.8, 121.8

[1,1'-Biphenyl]-2-ylmethanol (**3e**): [6]

Colorless oil; ^{1}H-NMR (400 MHz, $CDCl_3$): δ (ppm) 7.54-7.53 (m, 1H), 7.43-7.34 (m, 7H), 7.28-7.26 (m, 1H), 4.59 (s, 2H), 1.78 (b, 1H); ^{13}C-NMR (101 MHz, $CDCl_3$): δ (ppm) 141.3, 140.6, 139.1, 138.0, 130.0, 129.1, 128.3, 127.7, 127.6, 127.2, 63.1

(4'-Methoxy-[1,1'-biphenyl]-2-yl)methanol (3**f**): [7]

White solid, m.p. = 79-80°C, ^{1}H-NMR (400 MHz, $CDCl_3$): δ (ppm) 7.52 (dd, J = 6.9, 1.8Hz, 1H), 7.36-7.20 (m, 5H), 6.96-6.94 (m, 2H), 4.60 (s, 2H), 3.84 (s, 3H), 1.71

(s, 1H); ^{13}C-NMR (101 MHz, $CDCl_3$): δ (ppm) 158.7, 140.8, 132.8, 130.0, 130.0, 128.3, 127.5, 127.2, 113.5, 63.1, 55.1

(4'-(Trifluoromethyl)-[1,1'-biphenyl]-2-yl)methanol (**3g**): [8]

Light yellow oil, ^{1}H-NMR (400 MHz, $CDCl_3$): δ (ppm) 7.68 (d, J = 8Hz, 2H), 7.56 (dd, J = 7.5, 1.1Hz, 1H), 7.50 (d, J = 8Hz, 2H), 7.40 (dtd, 2H, J = 18.3, 7.4, 1.5Hz), 7.27 (dd, J = 7.4, 1.4Hz, 1H), 4.56 (s, 2H), 1.82 (s, 1H); ^{13}C-NMR (101 MHz, $CDCl_3$): δ (ppm) 144.3, 140.0, 137.7, 133.6, 129.9, 129.5, 128.7, 128.4, 127.9, 125.5, 125.2, 125.1, 125.1, 125.1, 62.9

(3'-Nitro-[1,1'-biphenyl]-2-yl)methanol (**3h**):

Yellow oil, ^{1}H-NMR (400 MHz, $CDCl_3$): δ (ppm) 8.28 (d, J = 1.7Hz, 1H), 8.23 (dd, J = 8.2, 1.1Hz, 1H), 7.76 (d, J = 7.6Hz, 1H), 7.59 (m, 2H), 7.44 (tdd, J = 15, 10.4, 4.3Hz, 2H), 7.30 (dd, J = 7.4, 1.2Hz, 1H), 4.58 (s, 2H), 1.94 (s, 1H); ^{13}C-NMR (101 MHz, $CDCl_3$): δ (ppm) 147.9, 142.1, 138.8, 137.5, 135.2, 132.9, 130.1, 129.8, 129.0, 128.9, 128.6, 128.0, 123.8, 123.1, 122.0, 121.9, 62.7

4-Nitro-1,1'-biphenyl (**3i**): [9]

Light yellow solid; m.p. 109-111°C; ^{1}H-NMR (400 MHz, $CDCl_3$): δ (ppm) 8.30 (m, 2H), 7.74 (m, 2H), 7.62 (m, 2H), 7.53 (m, 3H); ^{13}C-NMR (101 MHz, $CDCl_3$): δ (ppm) 147.6, 138.7, 129.1, 128.9, 127.8, 127.3, 124.1

4-Methoxy-4'-nitro-1,1'-biphenyl (**3j**): [7]

Light yellow solid, m.p. = 105-106°C, ^{1}H-NMR (400 MHz, $CDCl_3$): δ (ppm) 8.26 (d, J = 8.8Hz, 2H), 7.69 (d, J = 8.8Hz, 2H), 7.58 (d, J = 8.8Hz, 2H), 7.02 (d, J = 8.8Hz, 2H), 3.87 (s, 3H); ^{13}C-NMR (101 MHz, $CDCl_3$): δ (ppm) 160.2, 147.0, 146.3, 130.9, 128.4, 126.9, 124.0, 114.4, 55.2

4-Nitro-4'-(trifluoromethyl)-1,1'-biphenyl (**3k**): [10]

White solid, m.p.108-109°C, ^{1}H-NMR (400 MHz, $CDCl_3$): δ (ppm) 8.34 (d, J =8.8Hz, 2H), 7.75 (m, 6H); ^{13}C-NMR (101 MHz, $CDCl_3$): δ (ppm) 147.6, 146.0, 128.1, 127.8, 126.13, 126.09, 126.06, 126.02, 124.2

3,4'-Dinitro-1,1'-biphenyl (**3l**): [11]

Yellow solid, m.p. 184-187°C, ^{1}H-NMR (400 MHz, $CDCl_3$): δ (ppm) 8.50 (t, J = 1.8Hz, 1H), 8.37 (m, 2H), 8.31 (dd, J = 8.2, 2.1Hz, 1H), 7.96 (dd, J = 6.5, 1.3Hz, 1H), 7.80 (m, 2H), 7.71 (t, J = 8Hz, 1H); ^{13}C-NMR (101 MHz, $CDCl_3$): δ (ppm) 133.1, 130.1, 127.9, 124.3, 123.4, 122.1

[1,1'-Biphenyl]-2-ol (**3m**): [12]

White solid; m.p. 56-57°C, ^{1}H-NMR (400 MHz, $CDCl_3$): δ (ppm) 7.47 (m, 4H), 7.39 (m, 1H), 7.26 (m, 2H), 6.99 (td, J = 7.4, 1.0, 2H), 5.22 (s, 1H); ^{13}C-NMR (101 MHz,

$CDCl_3$): δ (ppm) 152.4, 137.1, 130.2, 129.2, 129.1, 128.7, 128.1, 127.8, 127.2, 127.1, 120.8, 115.8

4'-Methoxy-[1,1'-biphenyl]-2-ol (**3n**): [7]

Light yellow oil, ^{1}H-NMR (400 MHz, $CDCl_3$): δ (ppm) 7.40-7.38 (m, 2H), 7.25-7.20 (m, 2H), 7.02-6.95 (m, 4H), 5.25 (s, 1H), 3.45 (s, 3H); ^{13}C-NMR (101 MHz, $CDCl_3$): δ (ppm) 159.1, 152.3, 130.1, 129.0, 128.6, 127.6, 120.6, 115.5, 114.5, 55.2

4'-(Trifluoromethyl)-[1,1'-biphenyl]-2-ol (**3o**): [13]

White solid, m.p. 105-106°C, ^{1}H-NMR (400 MHz, $CDCl_3$): δ (ppm) 7.73 (d, J =8.2Hz, 2H), 7.63 (d, J =8.1Hz, 2H), 7.29 (m, 2H), 7.03 (t, J = 7.5Hz, 1H), 6.96 (d, J = 8.1Hz, 1H), 5.04 (s, 1H); ^{13}C-NMR (101 MHz, $CDCl_3$): δ (ppm) 152.3, 148.0, 141.1, 130.4, 129.8, 129.5, 126.9, 125.88, 125.84, 125.81, 125.77, 121.2, 116.2

3'-Nitro-[1,1'-biphenyl]-2-ol (**3p**):

^{1}H-NMR (400 MHz, $CDCl_3$): δ (ppm) 8.46 – 8.42 (m, 1H), 8.22 (ddd, J = 8.2, 2.3, 1.1 Hz, 1H), 7.89 (ddd, J = 7.7, 1.7, 1.1 Hz, 1H), 7.65 – 7.59 (m, 1H), 7.36 – 7.28 (m, 2H), 7.06 (td, J = 7.5, 1.1 Hz, 1H), 6.95 (dd, J = 8.0, 1.1 Hz, 1H), 5.07 (s, 1H) ; ^{13}C-NMR (101 MHz, $CDCl_3$): δ (ppm) 152.20, 139.40, 135.29, 133.03, 131.99, 129.96, 129.41, 129.15, 124.25, 123.27, 121.79, 116.10

[1,1'-Biphenyl]-4-carboxaldehyde (**3q**): [4]

White solid, m.p. = 58–60 °C; ^{1}H-NMR (400 MHz, $CDCl_3$): δ (ppm) 10.06 (s, 1H), 7.96 (m, 2H), 7.76 (m, 2H), 7.64 (m, 2H), 7.50 (dd, J = 11.5, 4.4Hz, 2H), 7.43 (d, J =7.3Hz, 1H); ^{13}C-NMR (101 MHz, $CDCl_3$): δ (ppm) 192.0, 147.0, 140.0, 135.0, 130.1, 128.9, 128.3, 127.5, 127.2

4'-Methoxy-[1,1'-biphenyl]-4-carboxaldehyde (**3r**): [14]

White solid, m.p. = 100-102°C, ^{1}H-NMR (400 MHz, $CDCl_3$): δ (ppm) 10.03 (s, 1H), 7.92 (d, J = 8.3Hz, 2H), 7.71 (d, J = 8.3Hz, 2H), 7.59 (d, J = 8.8Hz, 2H), 7.01 (d, J = 8.8Hz, 2H), 3.86 (s, 3H); ^{13}C-NMR (101 MHz, $CDCl_3$): δ (ppm) 191.7, 159.9, 146.6, 134.4, 131.8, 130.1, 128.3, 126.8, 114.3, 55.2

4'-(Trifluoromethyl)-[1,1'-biphenyl]-4-carboxaldehyde (**3s**): [15]

Colorless oil, ^{1}H-NMR (400 MHz, $CDCl_3$): δ (ppm) 10.08 (s, 1H), 7.99 (m, 2H), 7.76 (m, 6H); ^{13}C-NMR (101 MHz, $CDCl_3$): δ (ppm) 191.6, 145.4, 143.0, 135.6, 130.2, 127.8, 127.5, 125.8, 125.79, 125.76, 125.72

3'-Nitro-[1,1'-biphenyl]-4-carboxaldehyde (**3t**): [16]

Light yellow solid, m.p. 112-115°C, ^{1}H-NMR (400 MHz, $CDCl_3$): δ (ppm) 10.09 (s, 1H), 8.50 (t, J = 1.8Hz, 1H), 8.27 (dd, J =8.2, 1.2, 1H), 8.02 (d, J = 8.3Hz, 2H), 7.97 (d, J = 7.7Hz, 1H), 7.80 (d, J = 8.2Hz, 2H), 7.68 (t, J = 8Hz, 1H); ^{13}C-NMR (101 MHz, $CDCl_3$): δ (ppm) 191.6, 148.7, 144.3, 141.4, 133.2, 130.0, 127.8, 123.1, 122.2

[1,1'-Biphenyl]-4-amine (**3u**): [3]

Brown solid, m.p. = 49-51°C; ^{1}H-NMR (400 MHz, $CDCl_3$): δ (ppm) 7.53 (dd, J= 8.2, 1.3Hz, 2H), 7.29 (m, 4H), 7.26 (m, 1H), 6.74 (m, 2H), 3.70 (b, 2H); ^{13}C-NMR (101 MHz, $CDCl_3$): δ (ppm) 145.8, 141.0, 131.4, 128.5, 127.8, 126.2, 126.1, 115.2

4'-Methoxy-[1,1'-biphenyl]-4-amine (**3v**): [17]

Light yellow solid, m.p. = 143-145°C, ^{1}H-NMR (400 MHz, $CDCl_3$): δ (ppm) 7.48 (d, J = 8.9Hz, 2H), 7.38 (d, J = 8.6Hz, 2H), 6.96 (d, J = 8.9Hz, 2H), 6.75 (d, J = 8.6Hz, 2H), 3.85 (s, 3H), 3.70 (b, 2H); ^{13}C-NMR (101 MHz, $CDCl_3$): δ (ppm) 158.2, 145.1, 133.7, 131.1, 127.4, 127.2, 115.2, 113.9, 55.1

4'-(Trifluoromethyl)-[1,1'-biphenyl]-4-amine (**3w**): [18]

Yellow solid, m.p.140-144°C, ^{1}H-NMR (400 MHz, $CDCl_3$): δ (ppm) 7.62 (s, 4H), 7.42 (m, 2H), 6.75 (m, 2H), 3.79 (s, 2H); ^{13}C-NMR (101 MHz, $CDCl_3$): δ (ppm) 146.65, 144.57, 132.0, 129.7, 128.1, 126.4, 125.60, 125.57, 125.53, 125.49, 116.7, 115.3

3'-Nitro-[1,1'-biphenyl]-4-yl-amine (**3x**): [18]

Orange solid, m.p. 124-126°C, ^{1}H-NMR (400 MHz, $CDCl_3$): δ (ppm) 8.39 (t, J = 2Hz, 1H), 8.10 (ddd, J = 8.2, 2.3, 1.0Hz), 7.85 (ddd, J = 7.8, 1,8, 1.0Hz), 7.54 (t, J = 8Hz, 1H), 7.45 (m, 2H), 679 (m, 2H), 3.84 (s, 2H); ^{13}C-NMR (101 MHz, $CDCl_3$): δ (ppm) 146.8, 142.6, 131.9, 129.3, 128.5, 127.9, 115.3

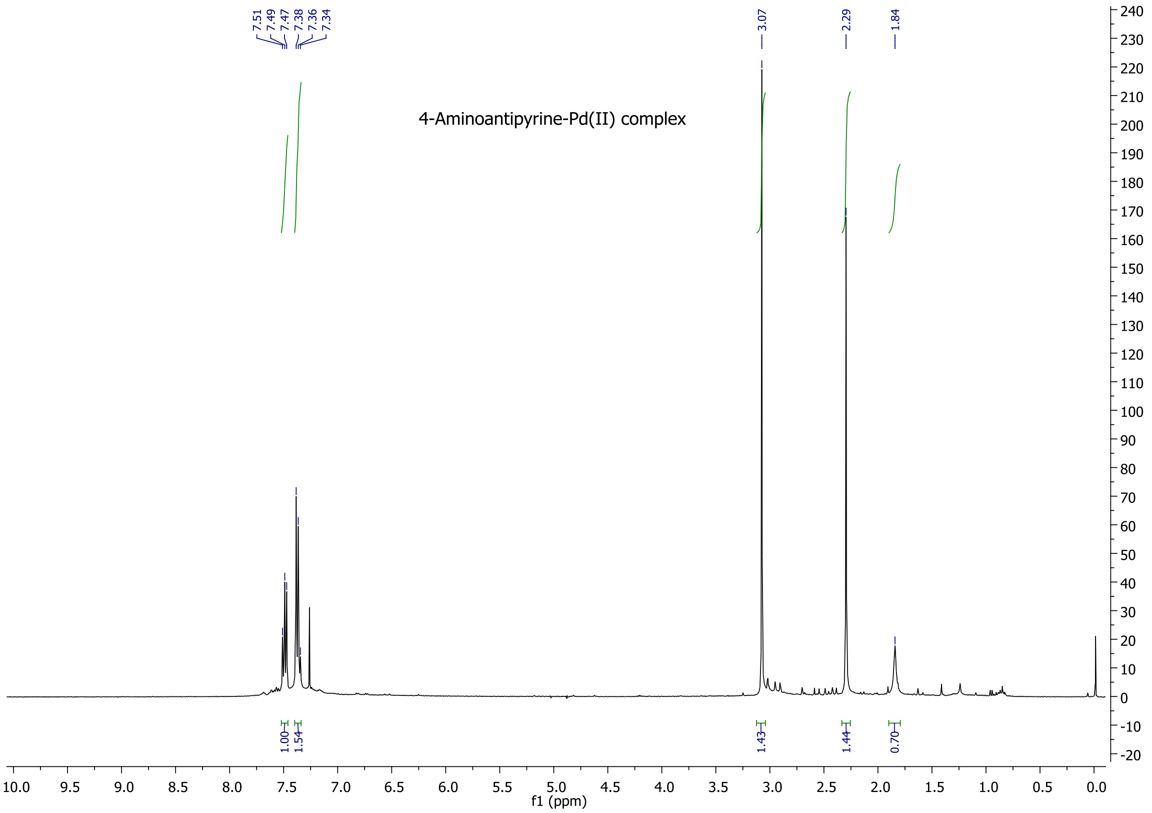
4-Aminoantipyrine-Pd(II) complex
7.51
7.49
7.47
7.38
7.36
7.34
3.07
2.29
1.84
1.00
1.54
1.43
1.44
0.70
f1 (ppm)

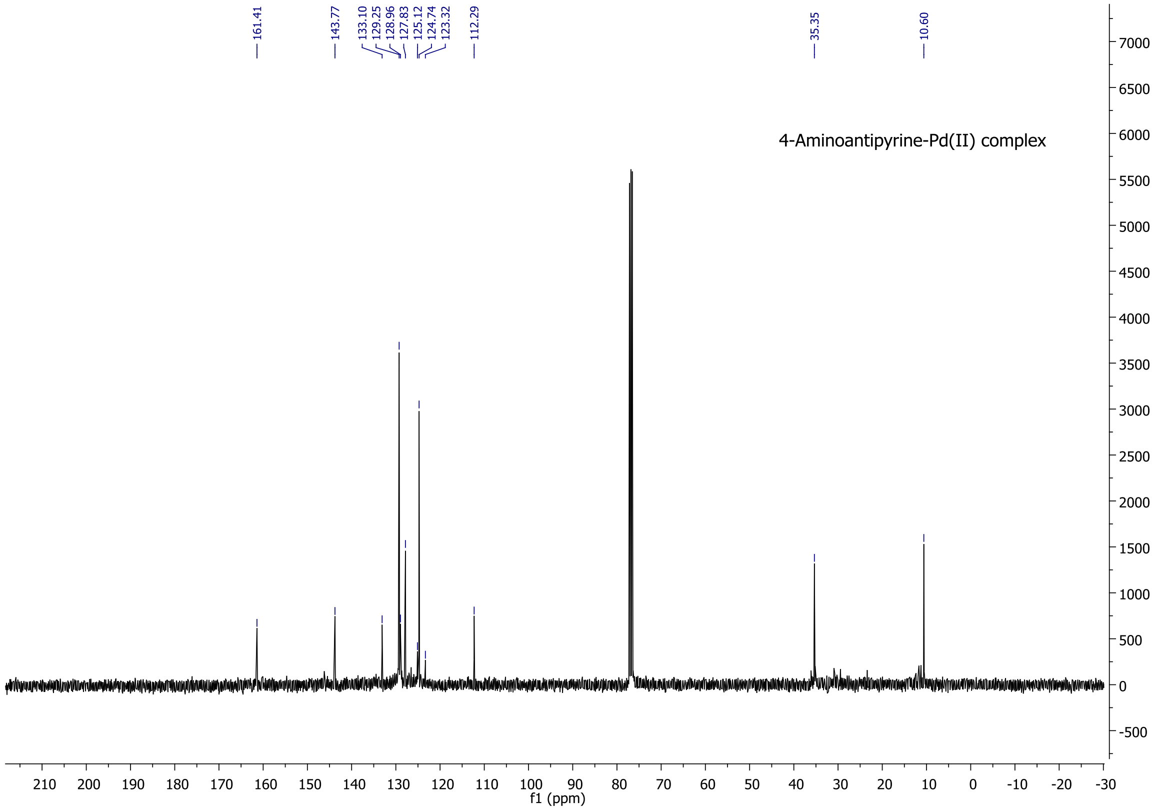
4-Aminoantipyrine-Pd(II) complex
161.41
143.77
133.10
129.25
128.96
127.83
125.12
124.74
123.32
112.29
35.35
10.60
f1 (ppm)

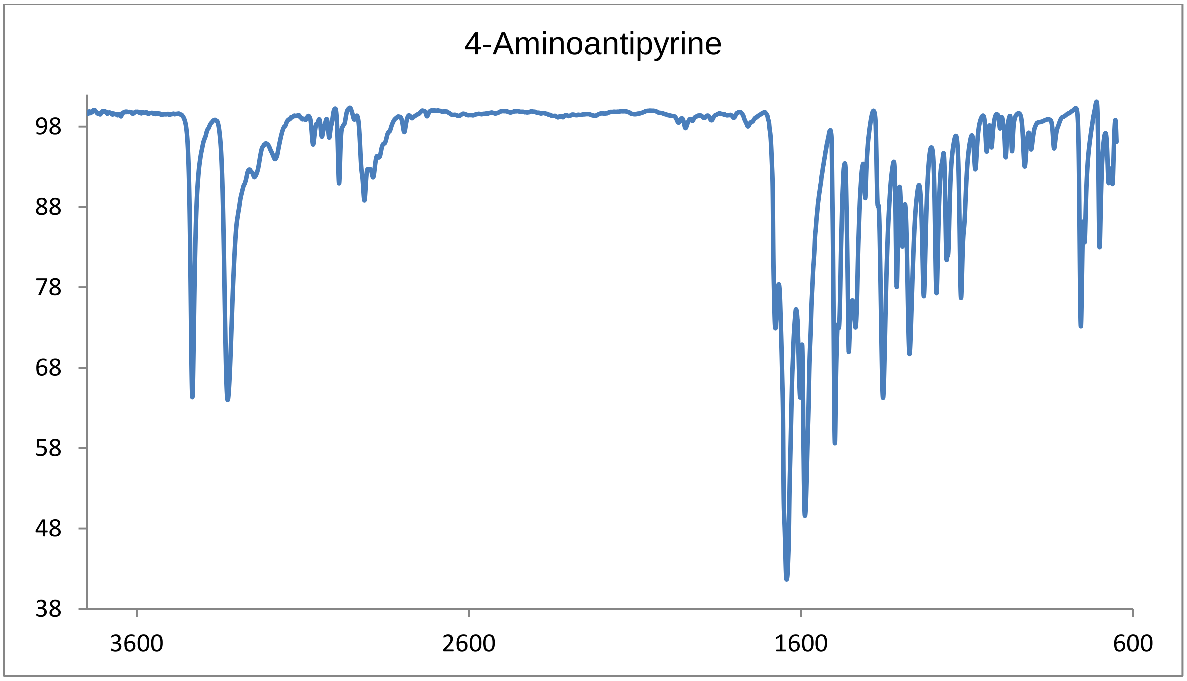
4-Aminoantipyrine
98
88
78
68
58
48
38
3600
2600
1600
600

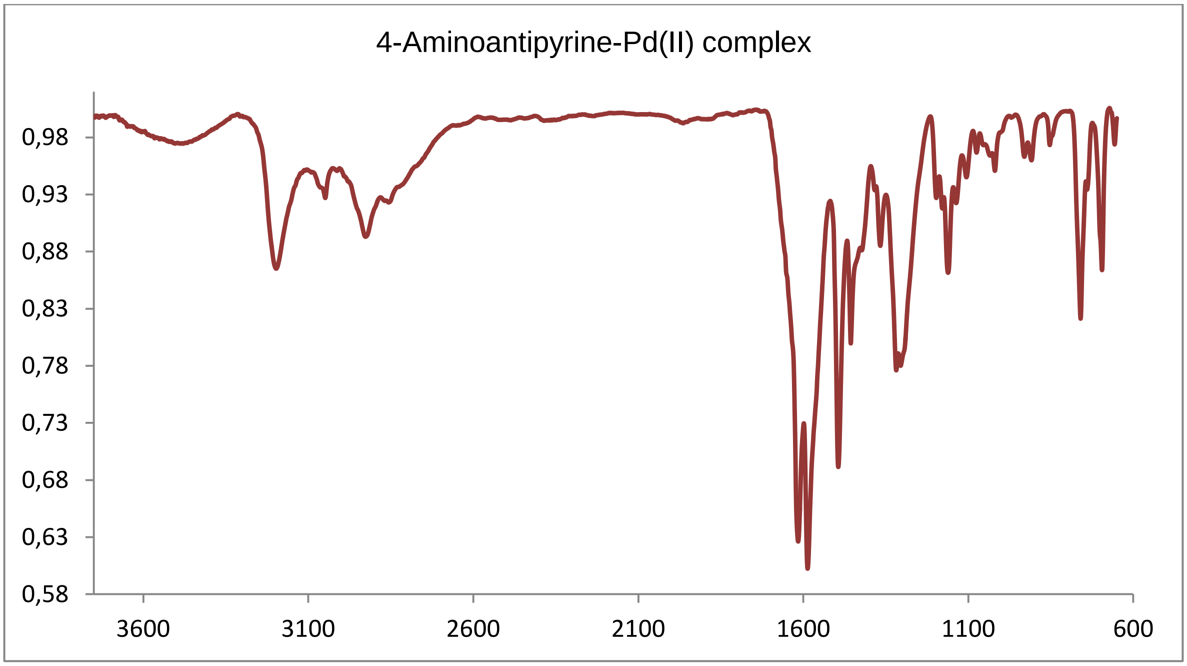
4-Aminoantipyrine-Pd(II) complex
0,98
0,93
0,88
0,83
0,78
0,73
0,68
0,63
0,58
3600
3100
2600
2100
1600
1100
600

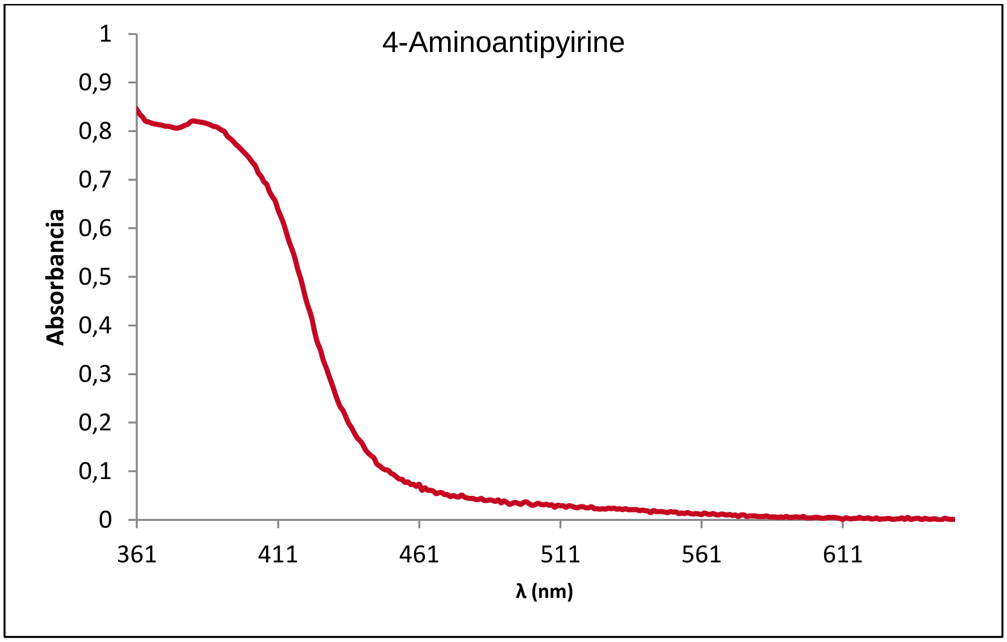

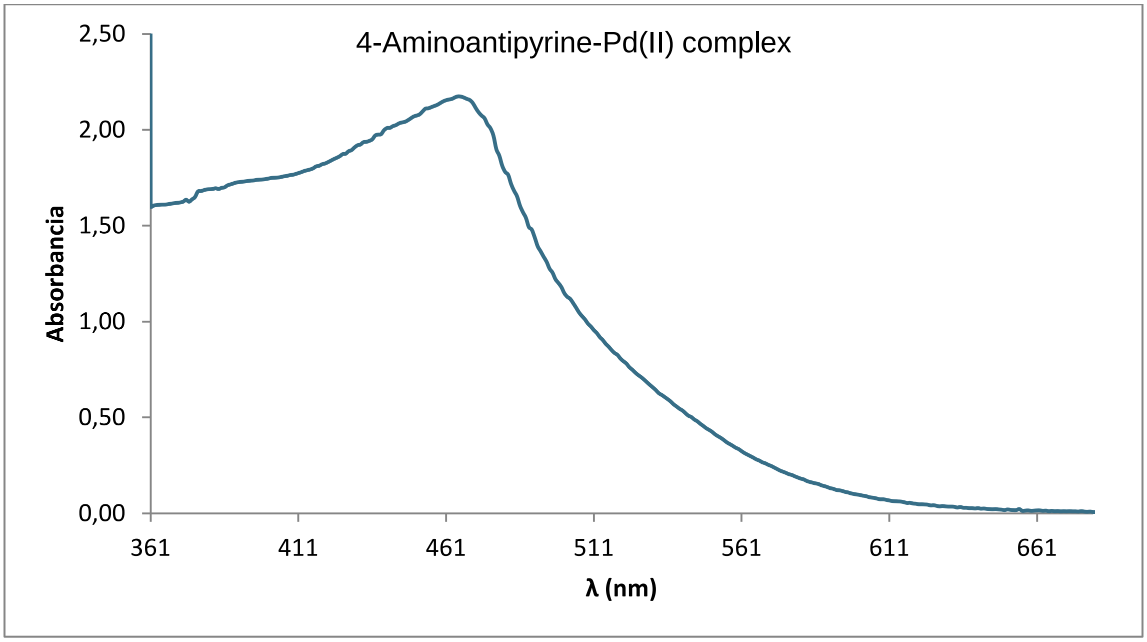

UV-Vis spectra, 1.5 mM in CH_3CN

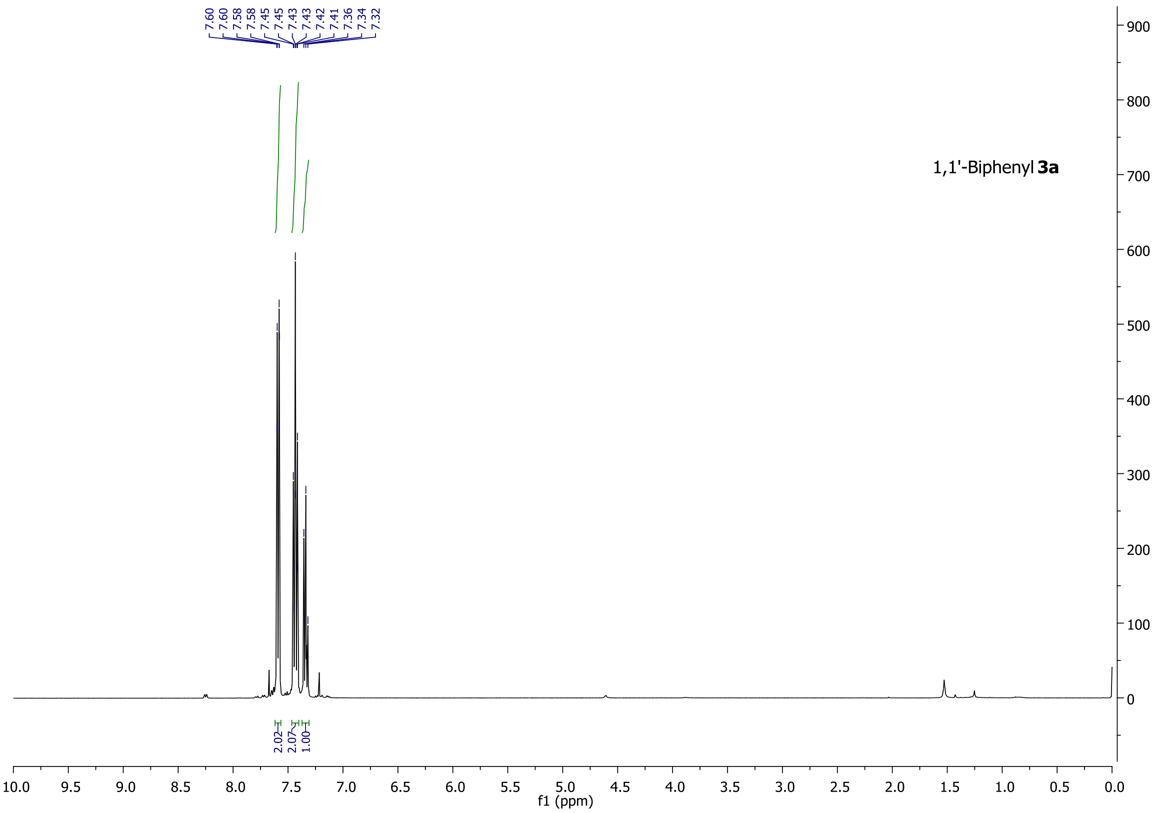

1,1'-Biphenyl **3a**

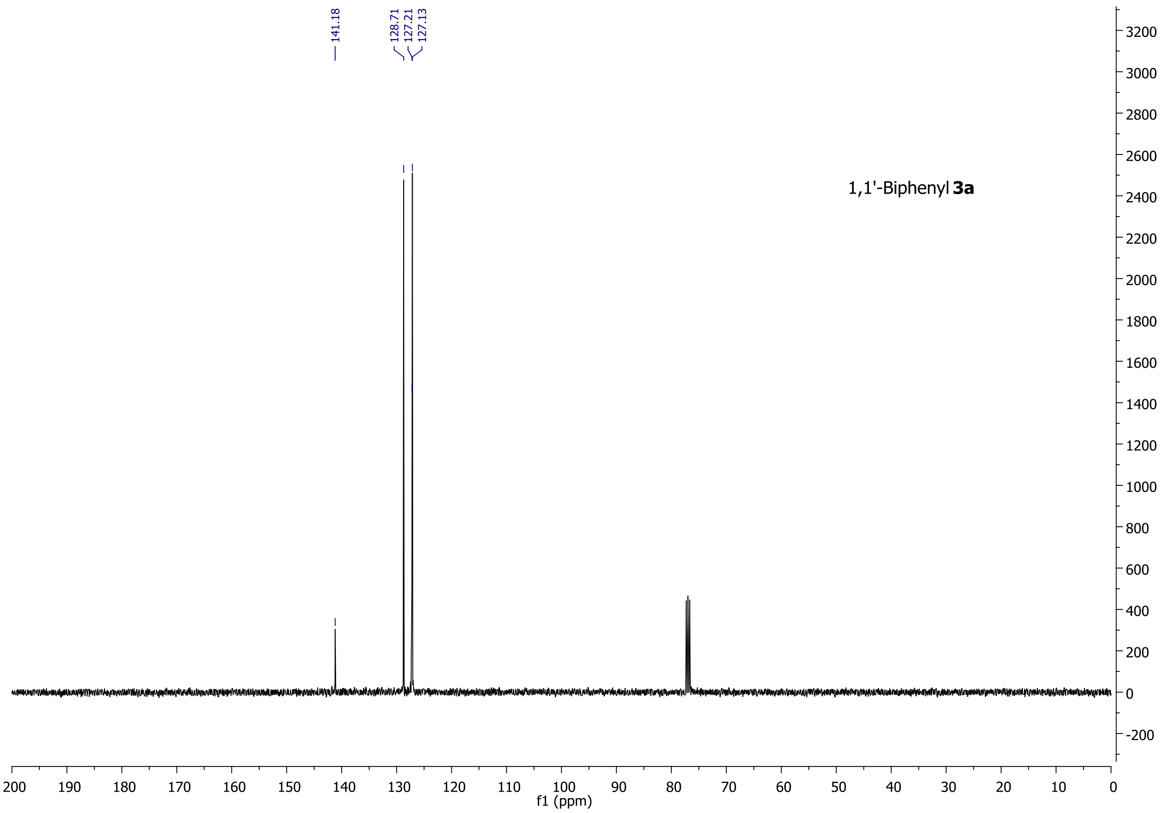

1,1'-Biphenyl **3a**

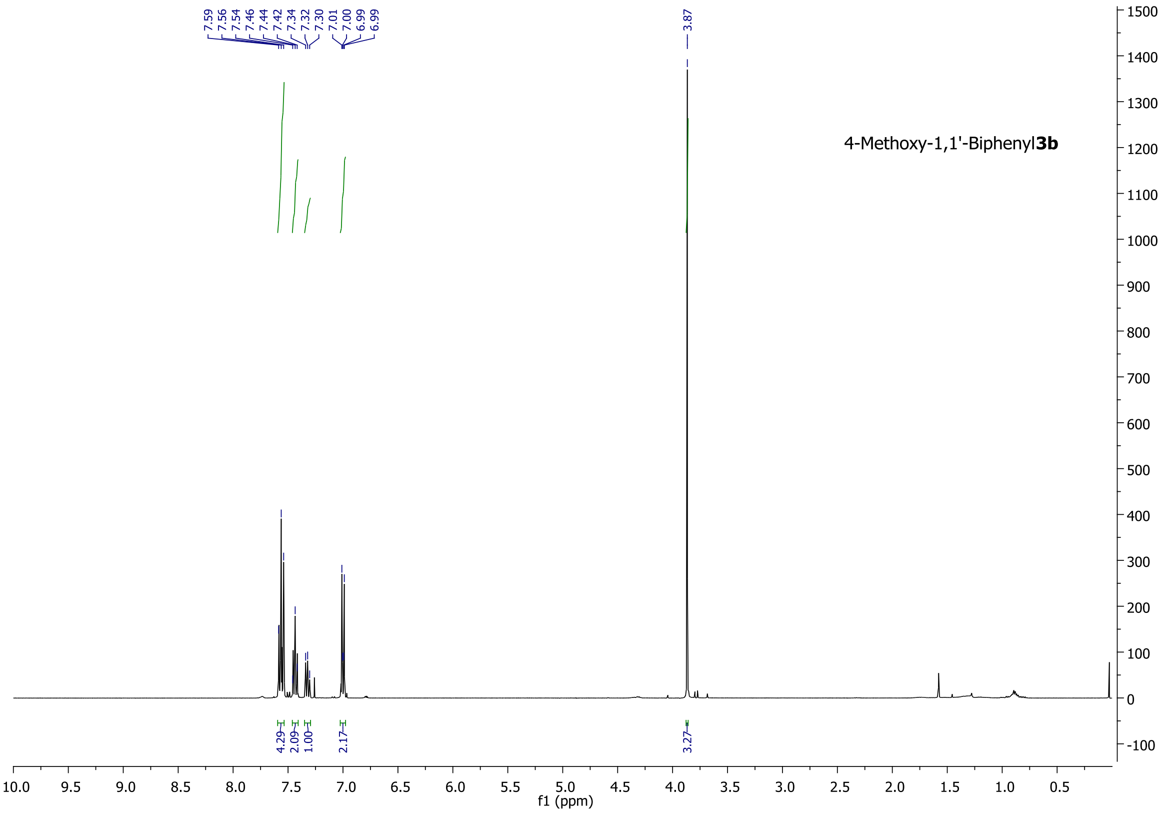
7.59
7.56
7.54
7.46
7.44
7.42
7.34
7.32
7.30
7.01
7.00
6.99
6.99
3.87
4-Methoxy-1,1'-Biphenyl3b
4.29
2.09
1.00
2.17
3.27
f1 (ppm)

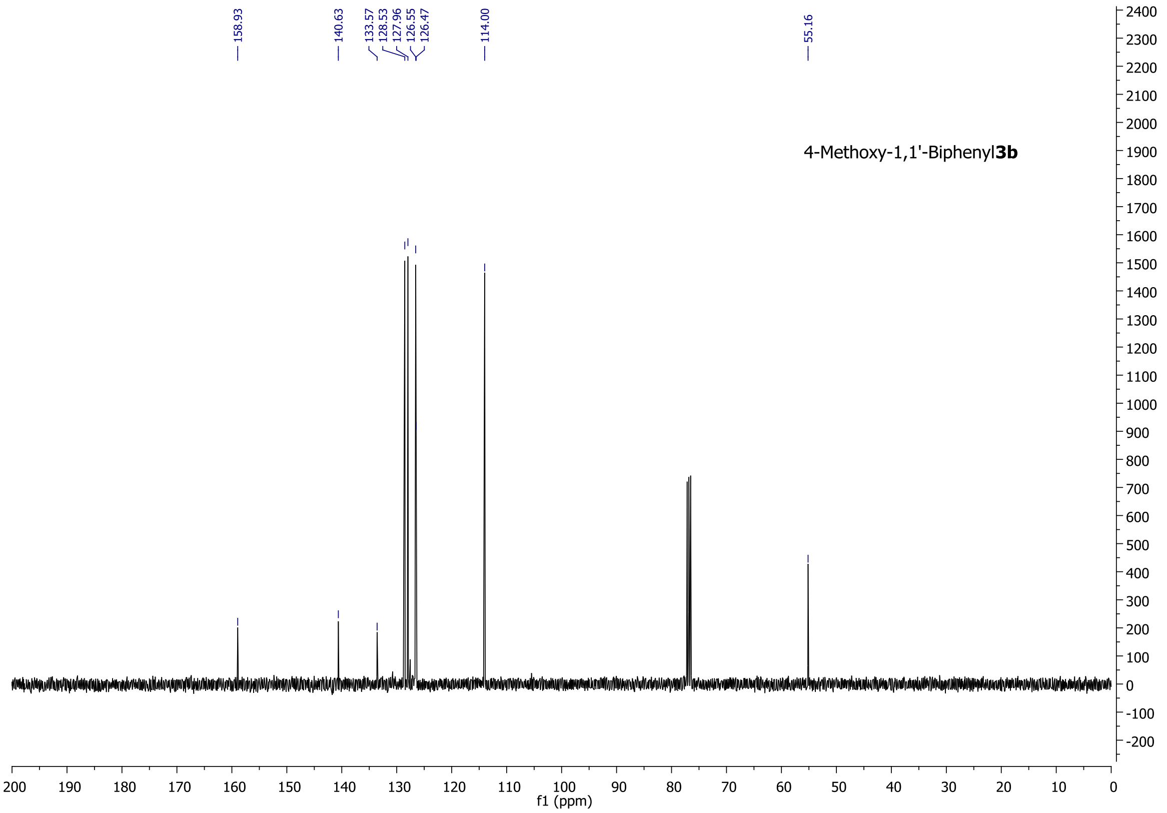
158.93
140.63
133.57
128.53
127.96
126.55
126.47
114.00
55.16
4-Methoxy-1,1'-Biphenyl3b
f1 (ppm)

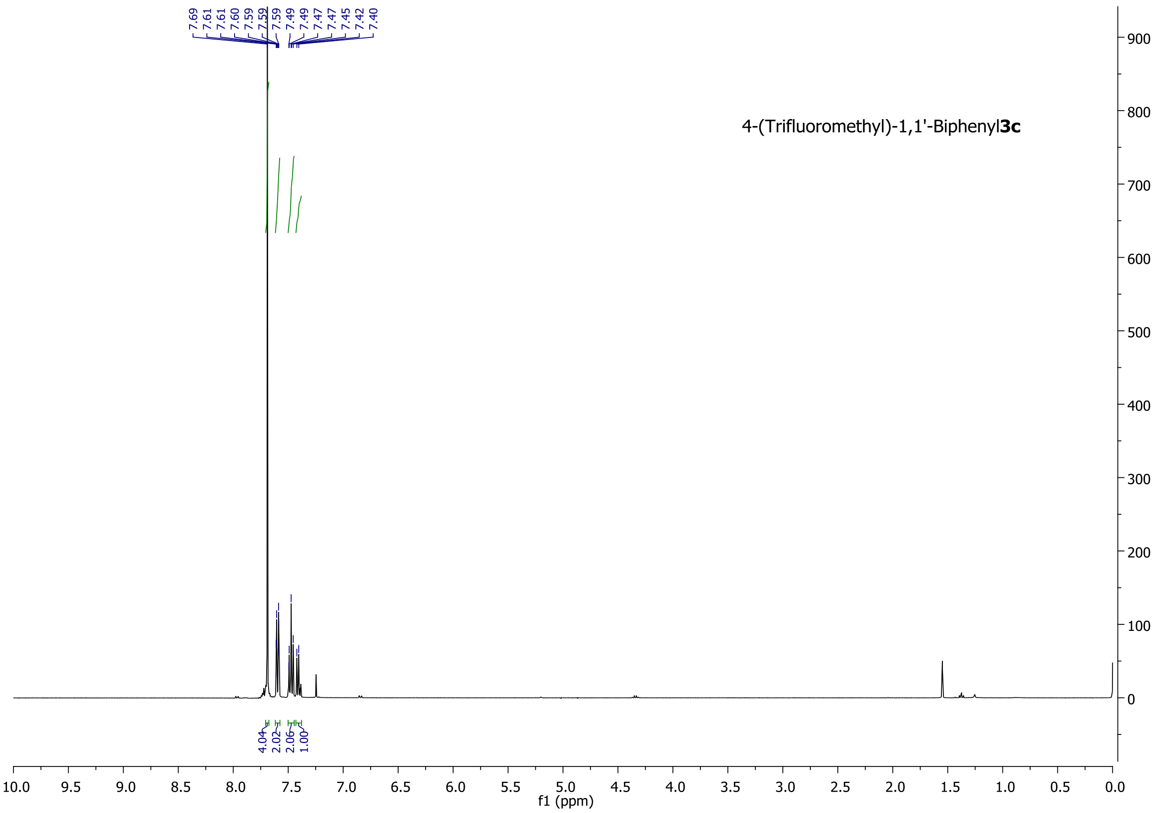

4-(Trifluoromethyl)-1,1'-Biphenyl**3c**

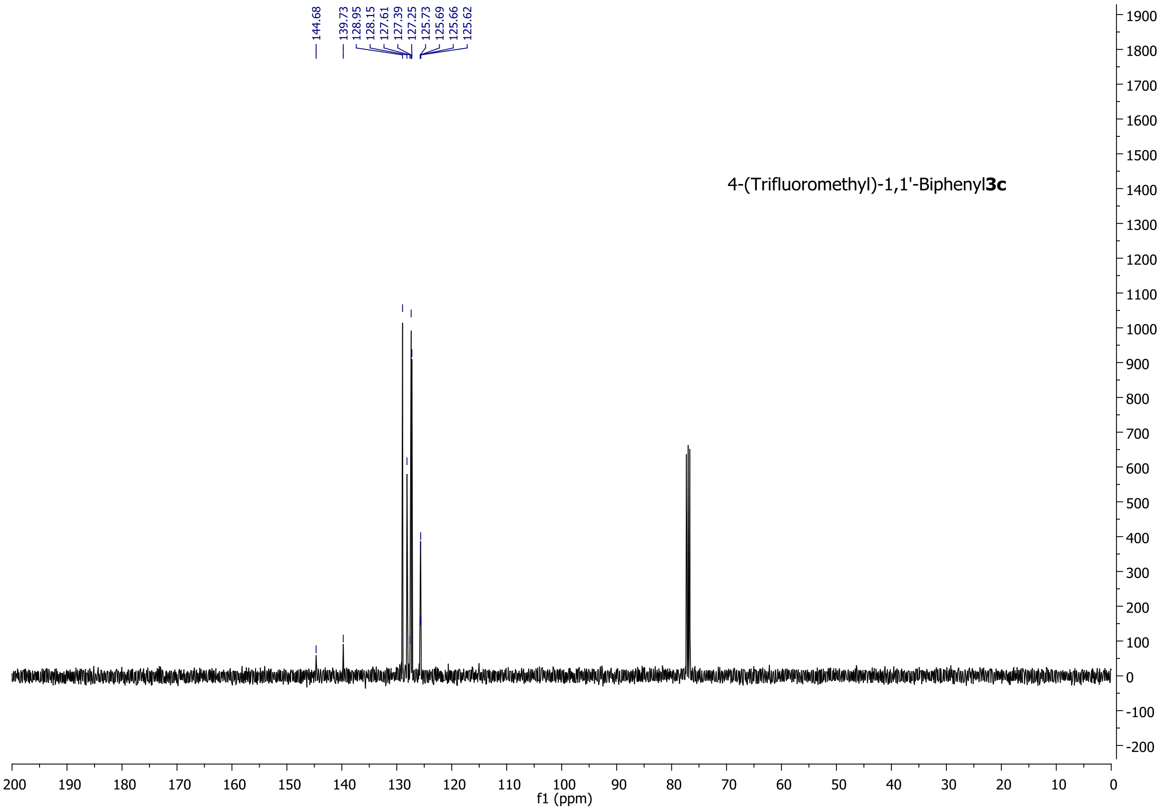

4-(Trifluoromethyl)-1,1'-Biphenyl**3c**

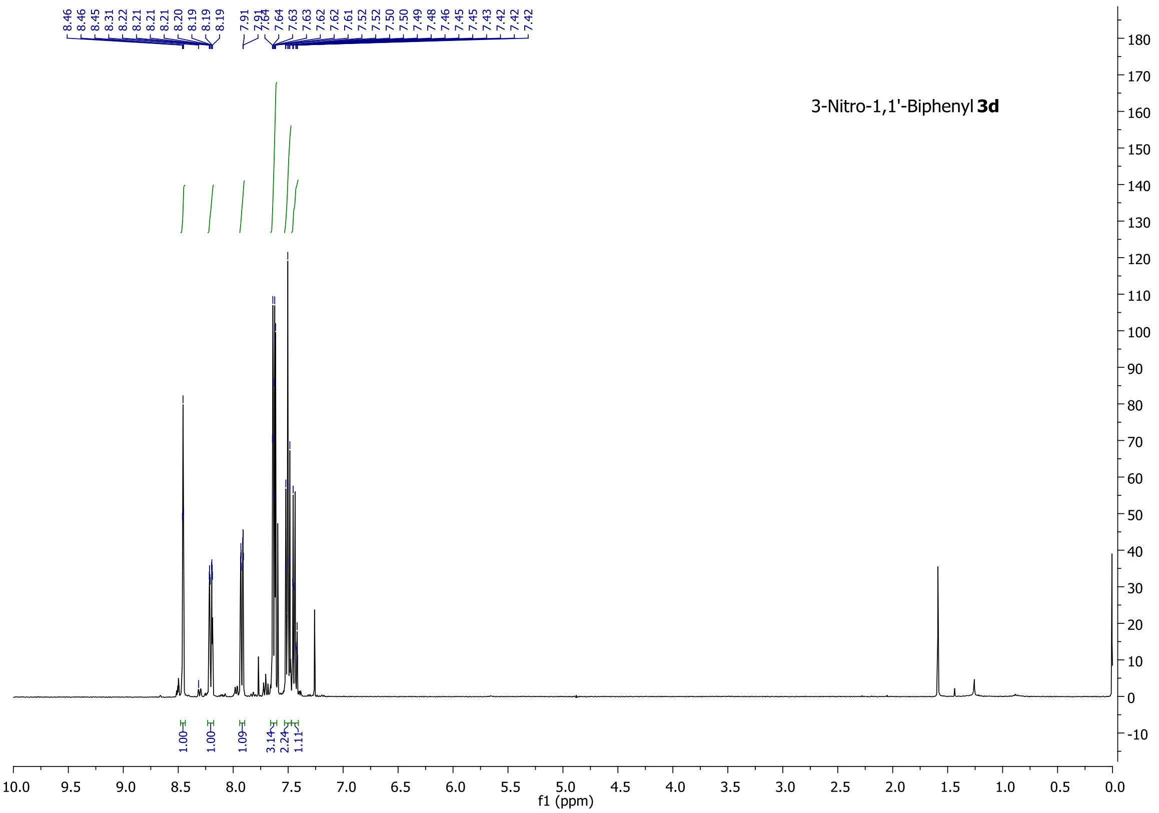
3-Nitro-1,1'-Biphenyl **3d**
f1 (ppm)

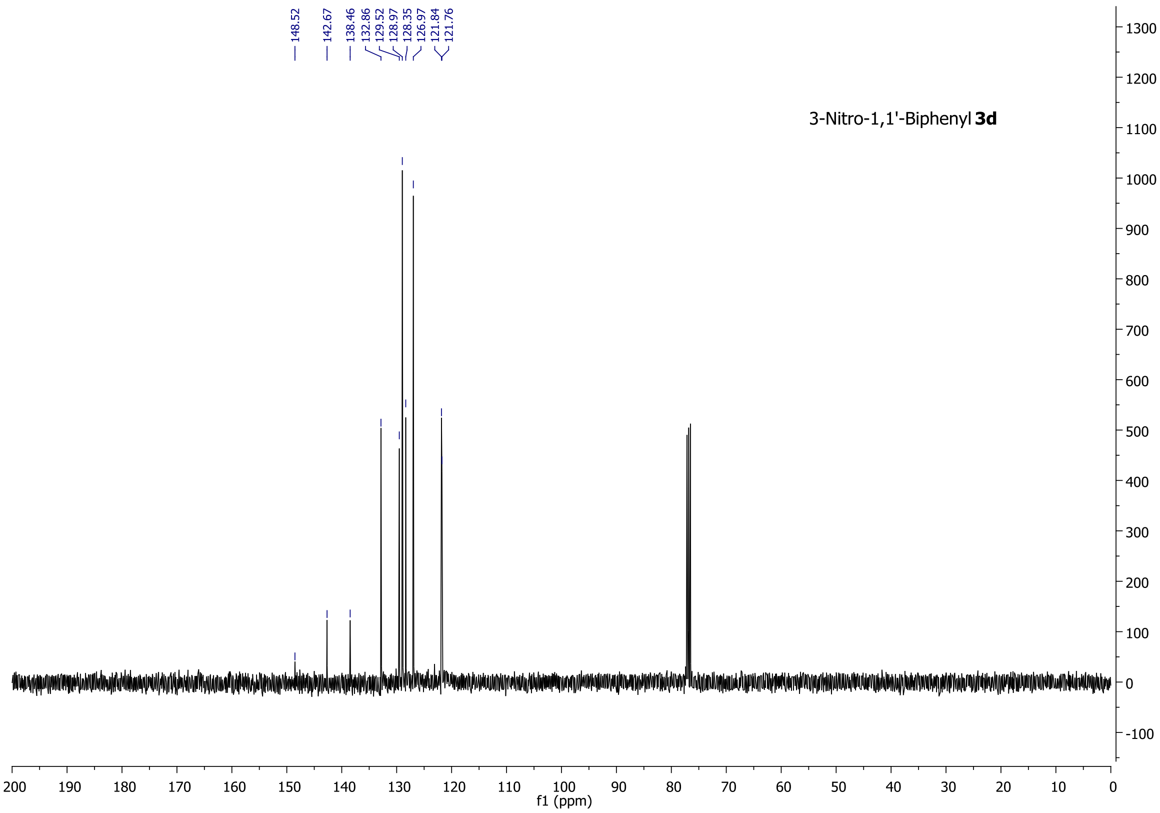
148.52
142.67
138.46
132.86
129.52
128.97
128.35
126.97
121.84
121.76
3-Nitro-1,1'-Biphenyl **3d**
f1 (ppm)

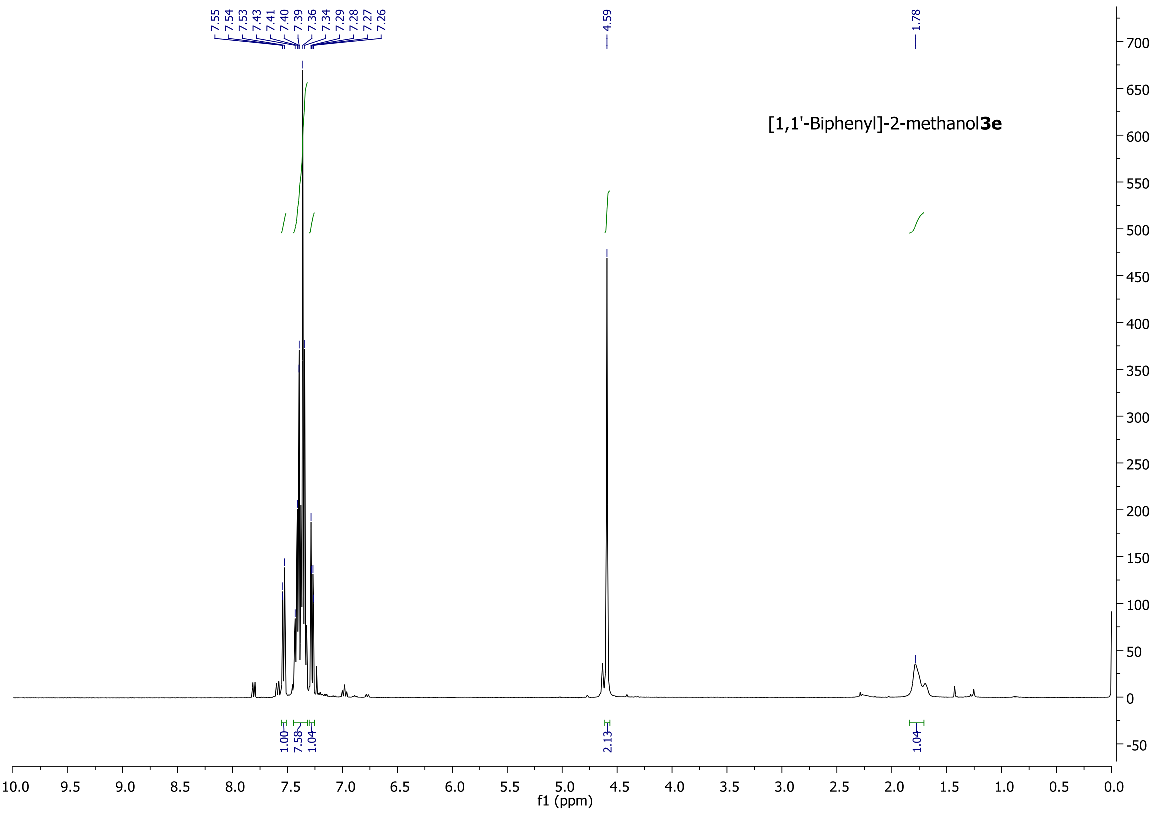

7.55
7.54
7.53
7.43
7.41
7.40
7.39
7.36
7.34
7.29
7.28
7.27
7.26
4.59
1.78
[1,1'-Biphenyl]-2-methanol**3e**
1.00
7.58
1.04
2.13
1.04
f1 (ppm)

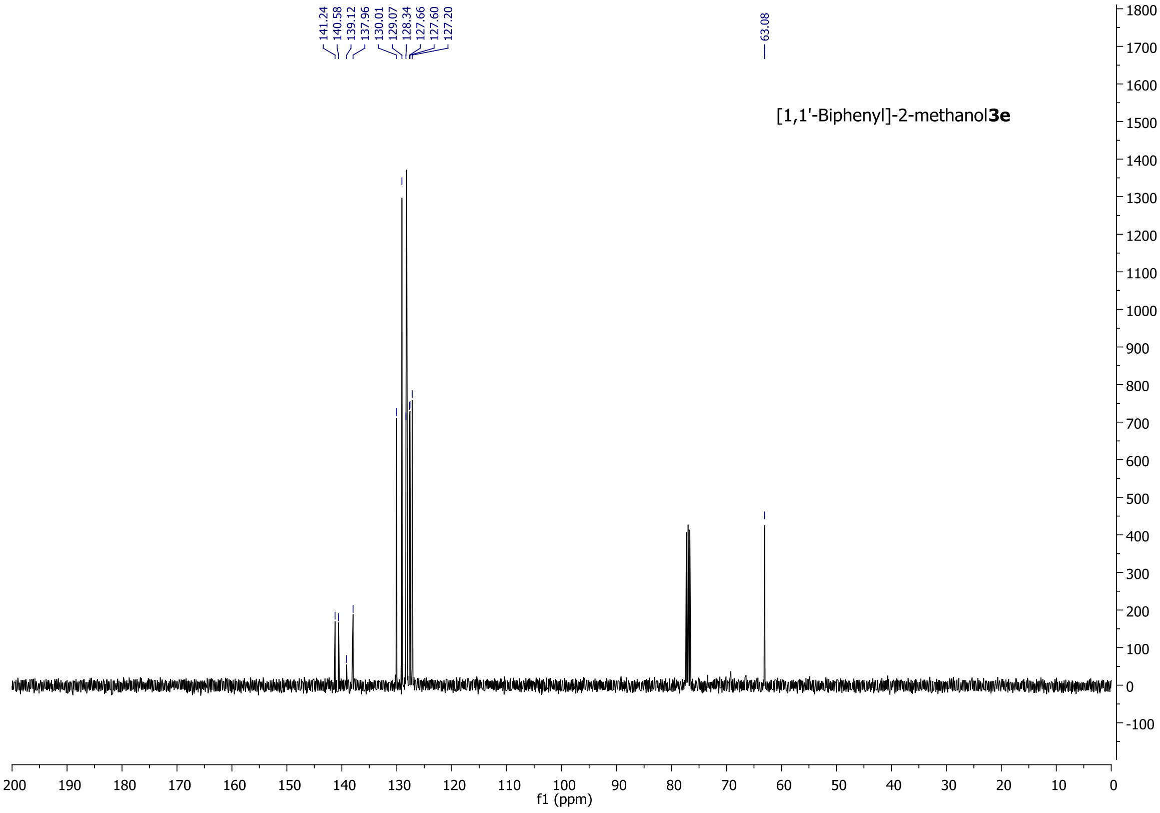

141.24
140.58
139.12
137.96
130.01
129.07
128.34
127.66
127.60
127.20
63.08
[1,1'-Biphenyl]-2-methanol**3e**
f1 (ppm)

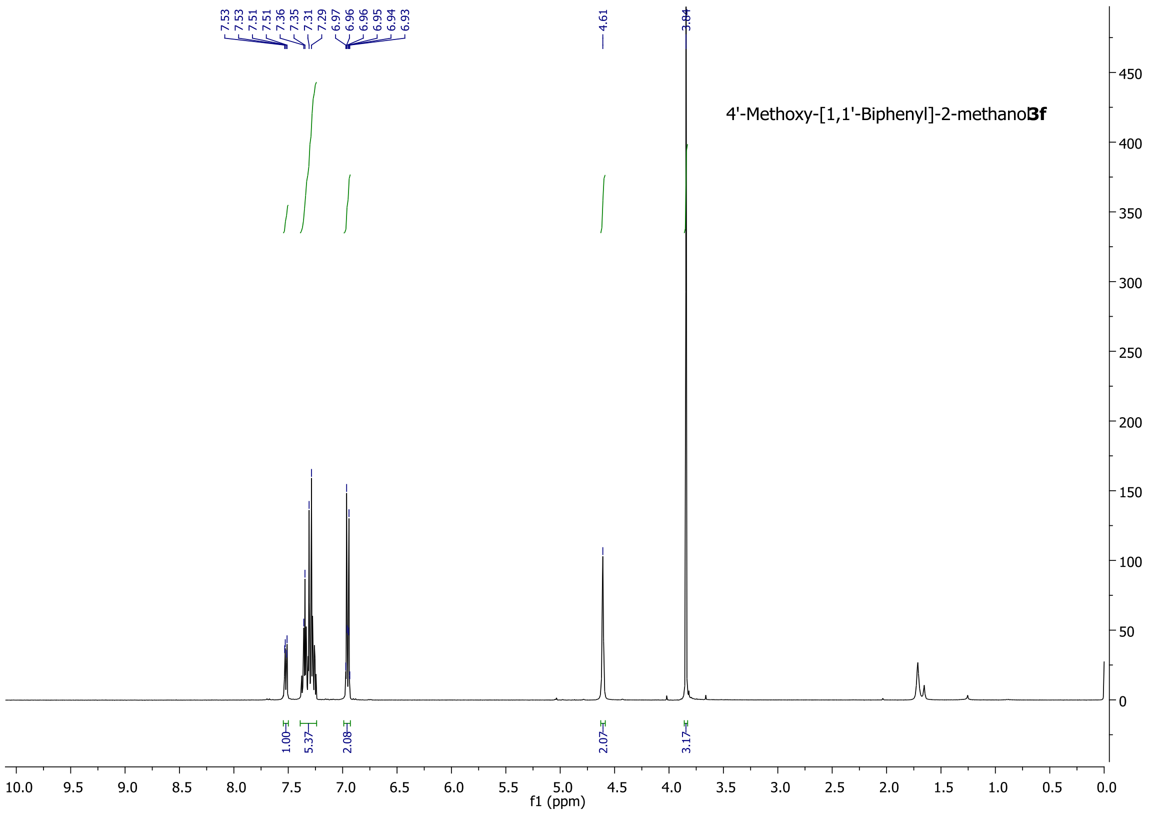
4'-Methoxy-[1,1'-Biphenyl]-2-methanol **3f**
f1 (ppm)

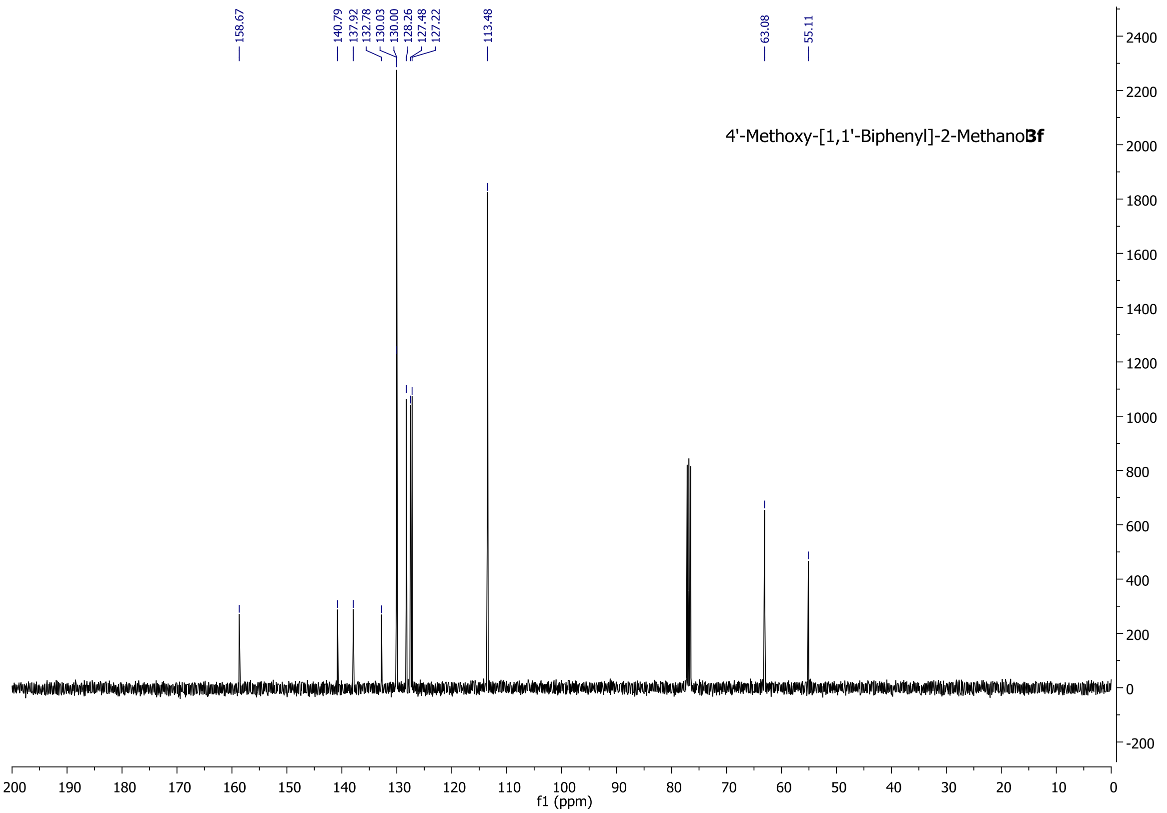
4'-Methoxy-[1,1'-Biphenyl]-2-Methanol **3f**
f1 (ppm)

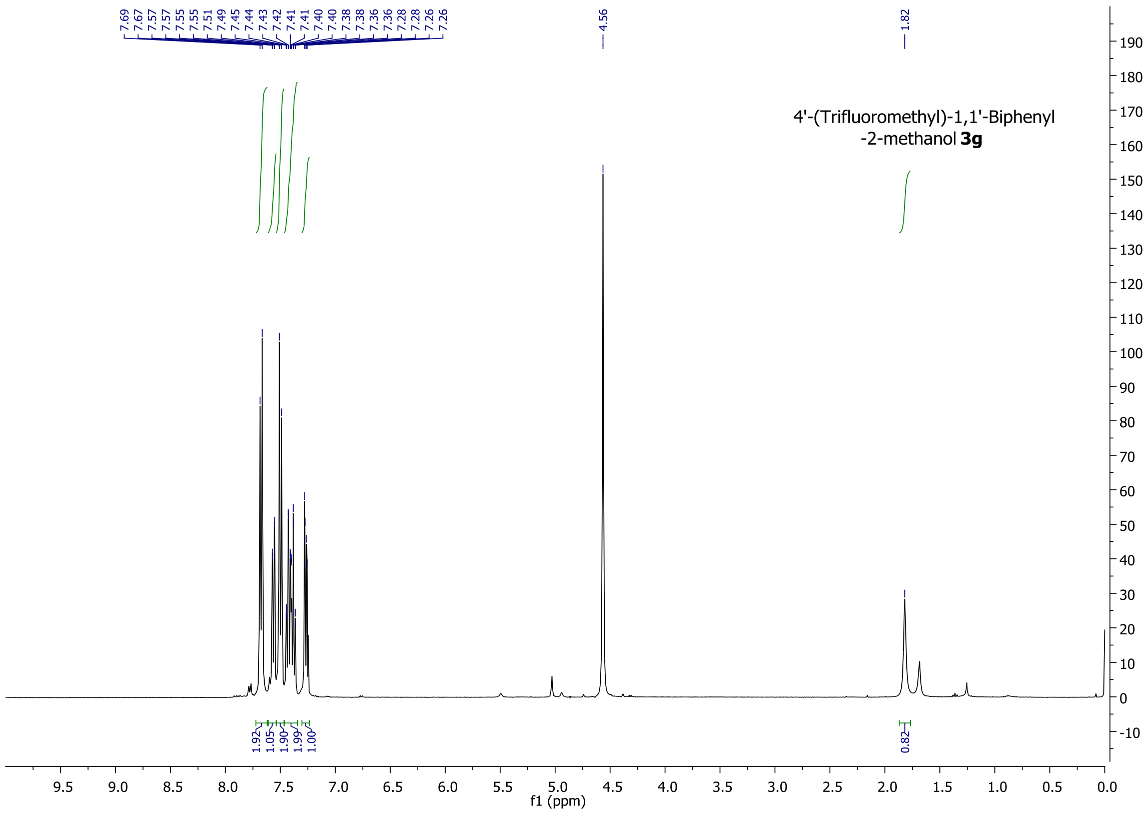
7.69 7.67 7.57 7.57 7.55 7.55 7.51 7.49 7.45 7.44 7.43 7.42 7.41 7.41 7.40 7.40 7.38 7.38 7.36 7.36 7.28 7.28 7.26 7.26
4.56
1.82
4'-(Trifluoromethyl)-1,1'-Biphenyl
-2-methanol **3g**
1.92 1.05 1.90 1.99 1.00
0.82
f1 (ppm)

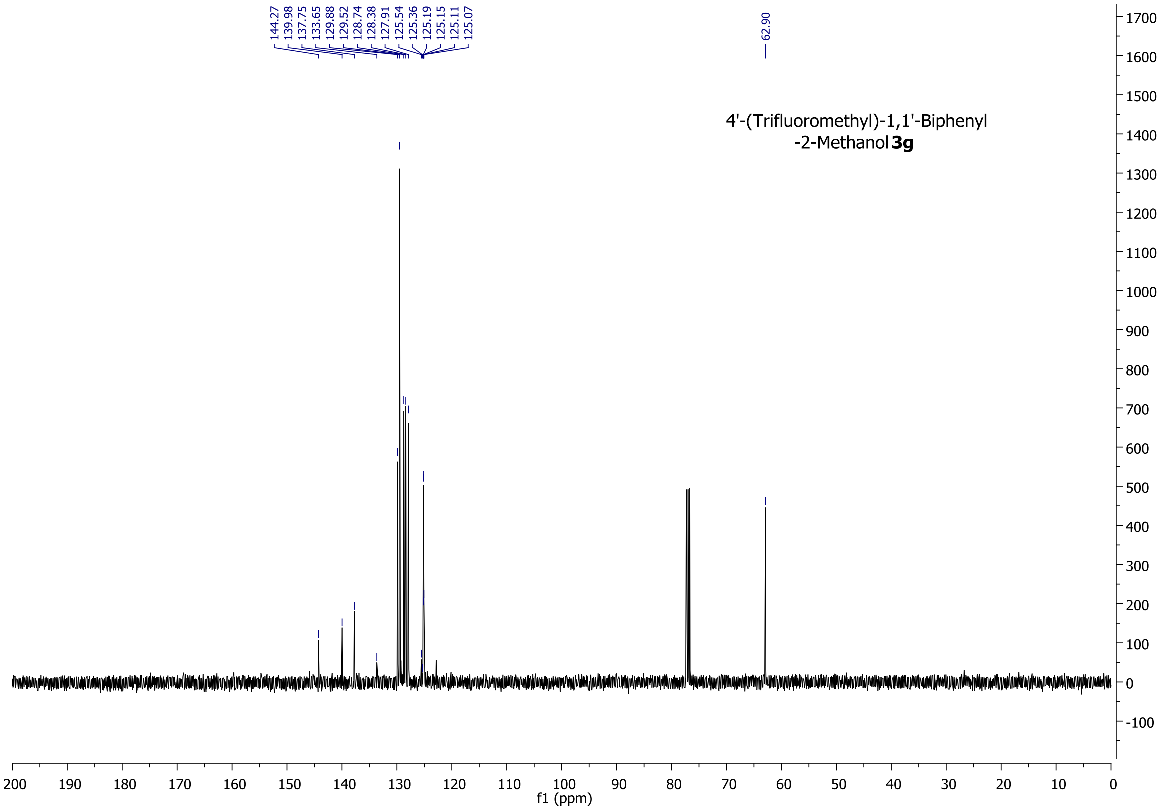
144.27 139.98 137.75 133.65 129.88 129.52 128.74 128.38 127.91 125.54 125.36 125.19 125.15 125.11 125.07
62.90
4'-(Trifluoromethyl)-1,1'-Biphenyl
-2-Methanol **3g**
f1 (ppm)

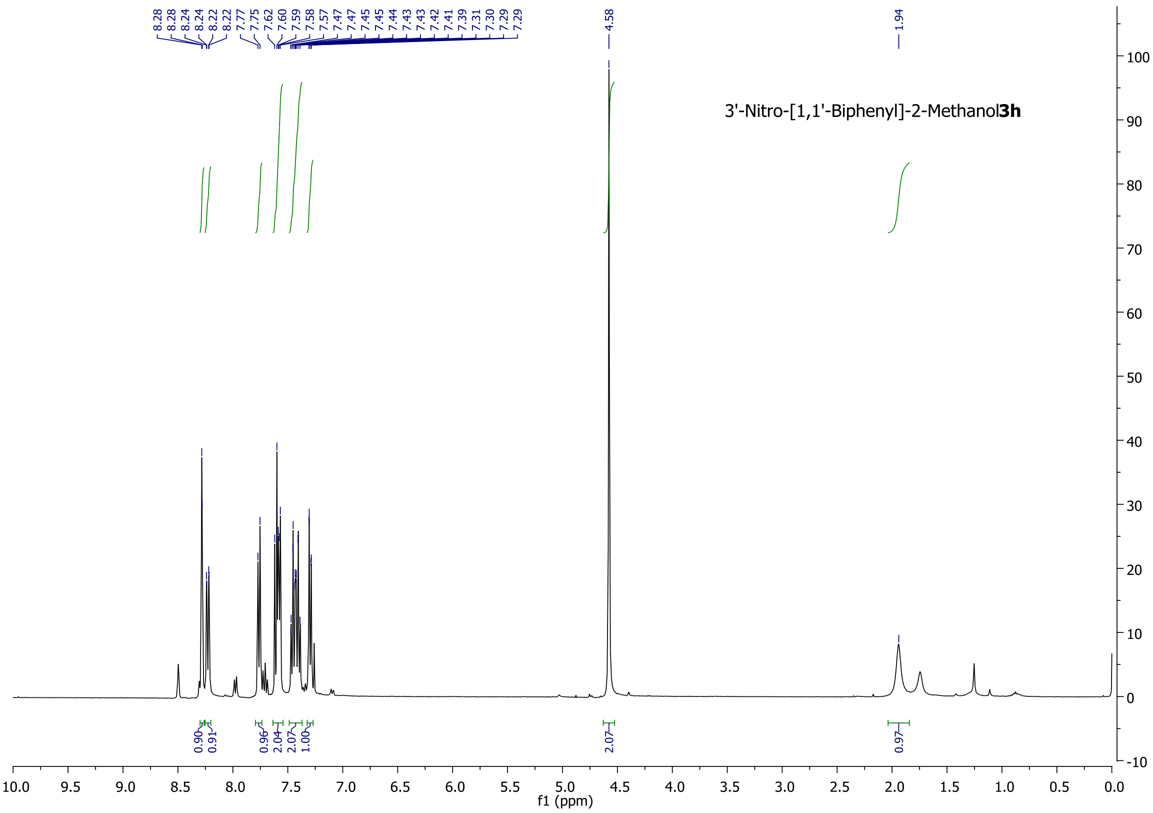
8.28
8.28
8.24
8.24
8.22
8.22
7.77
7.75
7.62
7.60
7.59
7.58
7.57
7.47
7.47
7.45
7.44
7.43
7.43
7.42
7.41
7.39
7.31
7.30
7.29
7.29
4.58
1.94
3'-Nitro-[1,1'-Biphenyl]-2-Methanol**3h**
0.90
0.91
0.96
2.04
2.07
1.00
2.07
0.97
f1 (ppm)

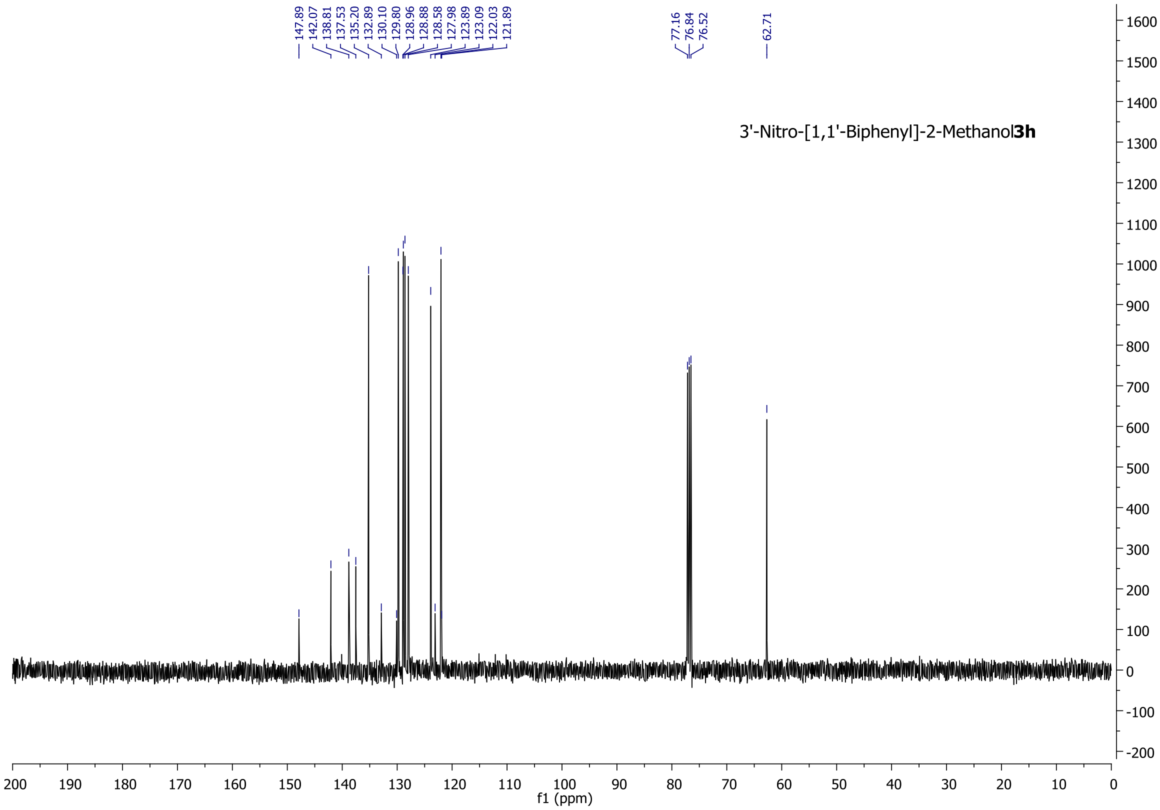
147.89
142.07
138.81
137.53
135.20
132.89
130.10
129.80
128.96
128.88
128.58
127.98
123.89
123.09
122.03
121.89
77.16
76.84
76.52
62.71
3'-Nitro-[1,1'-Biphenyl]-2-Methanol**3h**
f1 (ppm)

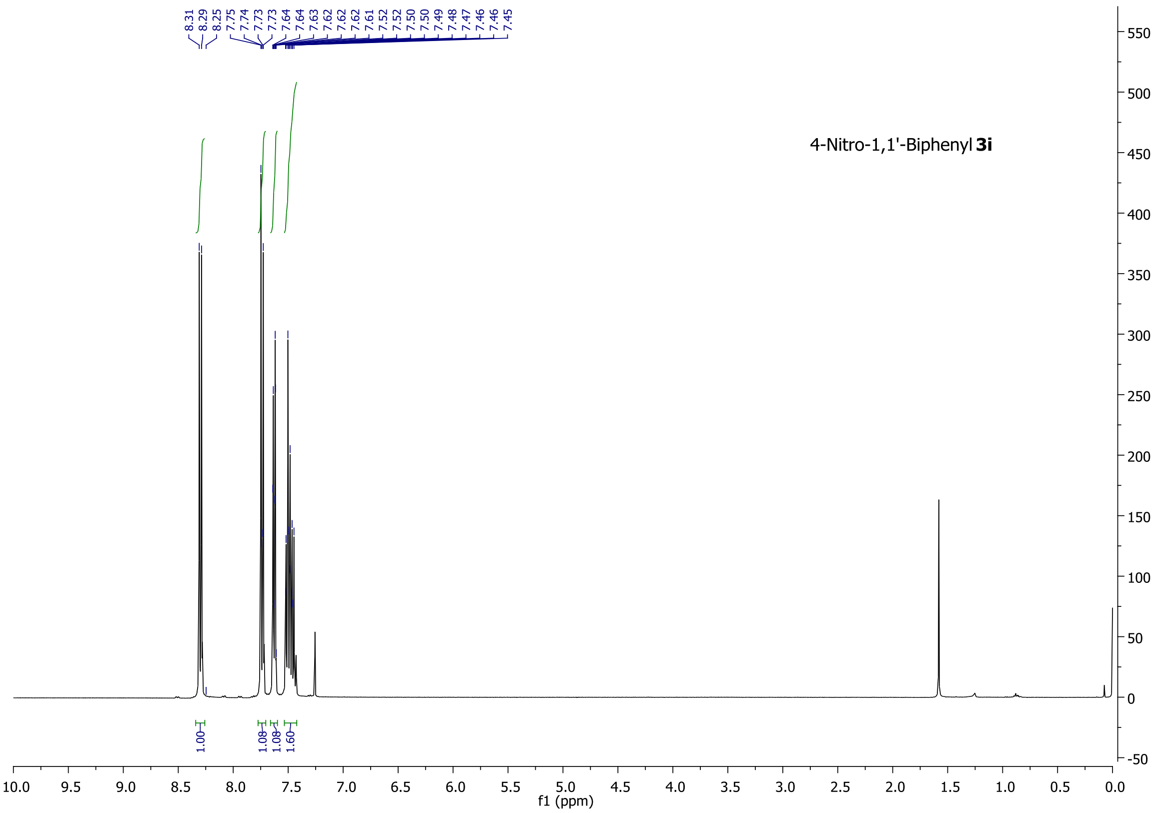
4-Nitro-1,1'-Biphenyl **3i**
8.31
8.29
8.25
7.75
7.74
7.73
7.73
7.64
7.64
7.63
7.62
7.62
7.61
7.52
7.52
7.50
7.50
7.49
7.48
7.47
7.46
7.45
1.00
1.08
1.08
1.60
f1 (ppm)

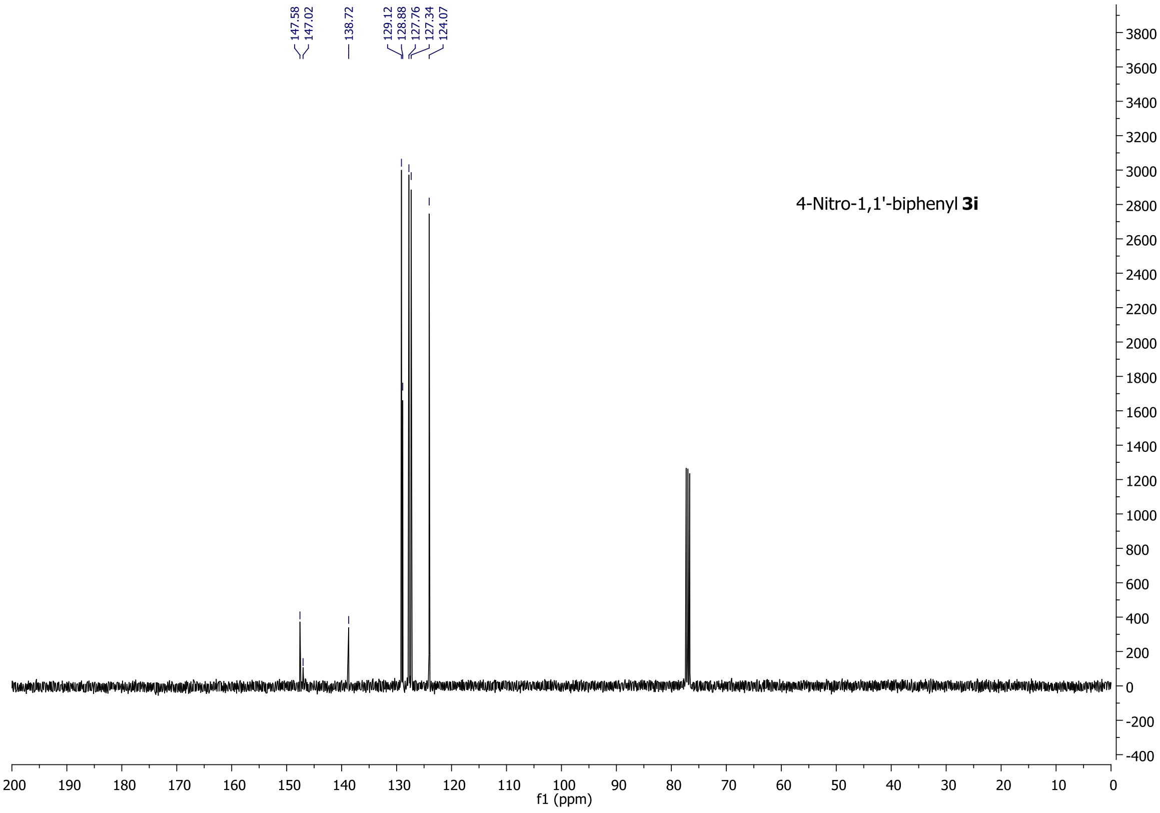
4-Nitro-1,1'-biphenyl **3i**
147.58
147.02
138.72
129.12
128.88
127.76
127.34
124.07
f1 (ppm)

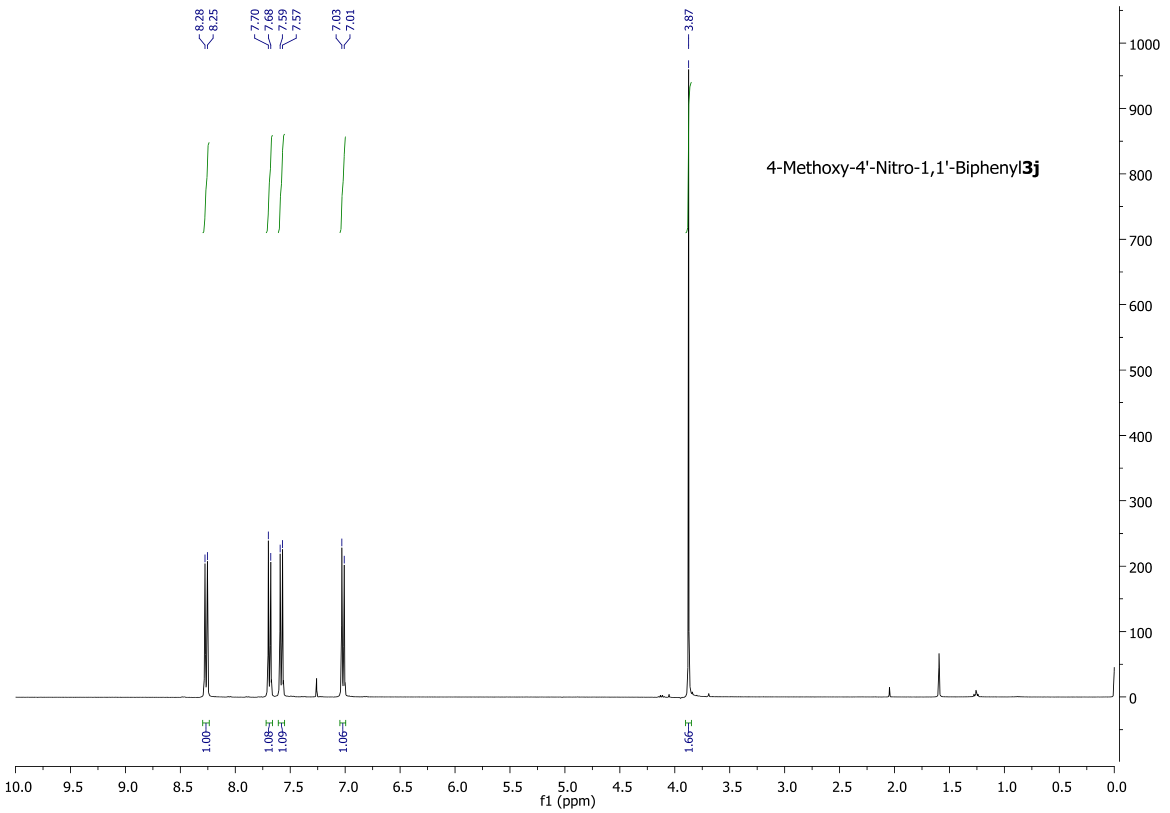

8.28
8.25
7.70
7.68
7.59
7.57
7.03
7.01
3.87
4-Methoxy-4'-Nitro-1,1'-Biphenyl**3j**
1.00
1.08
1.09
1.06
1.66
f1 (ppm)

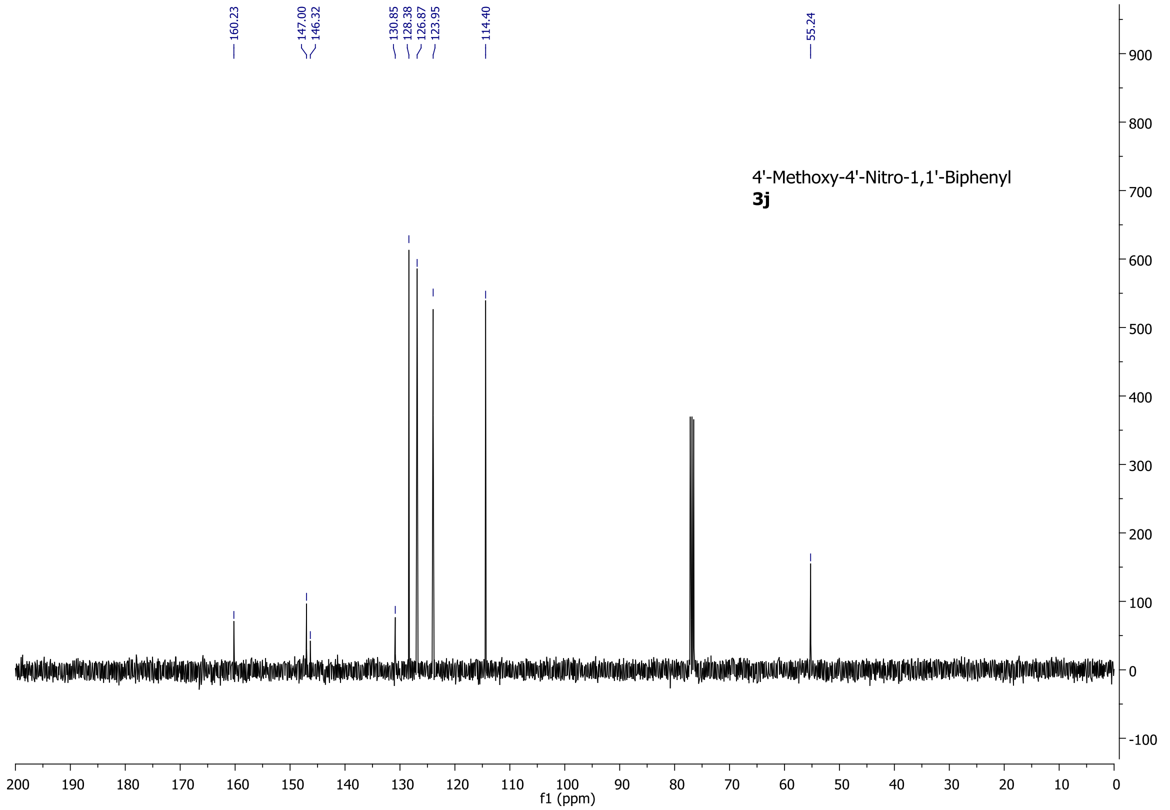

160.23
147.00
146.32
130.85
128.38
126.87
123.95
114.40
55.24
4'-Methoxy-4'-Nitro-1,1'-Biphenyl
3j
f1 (ppm)

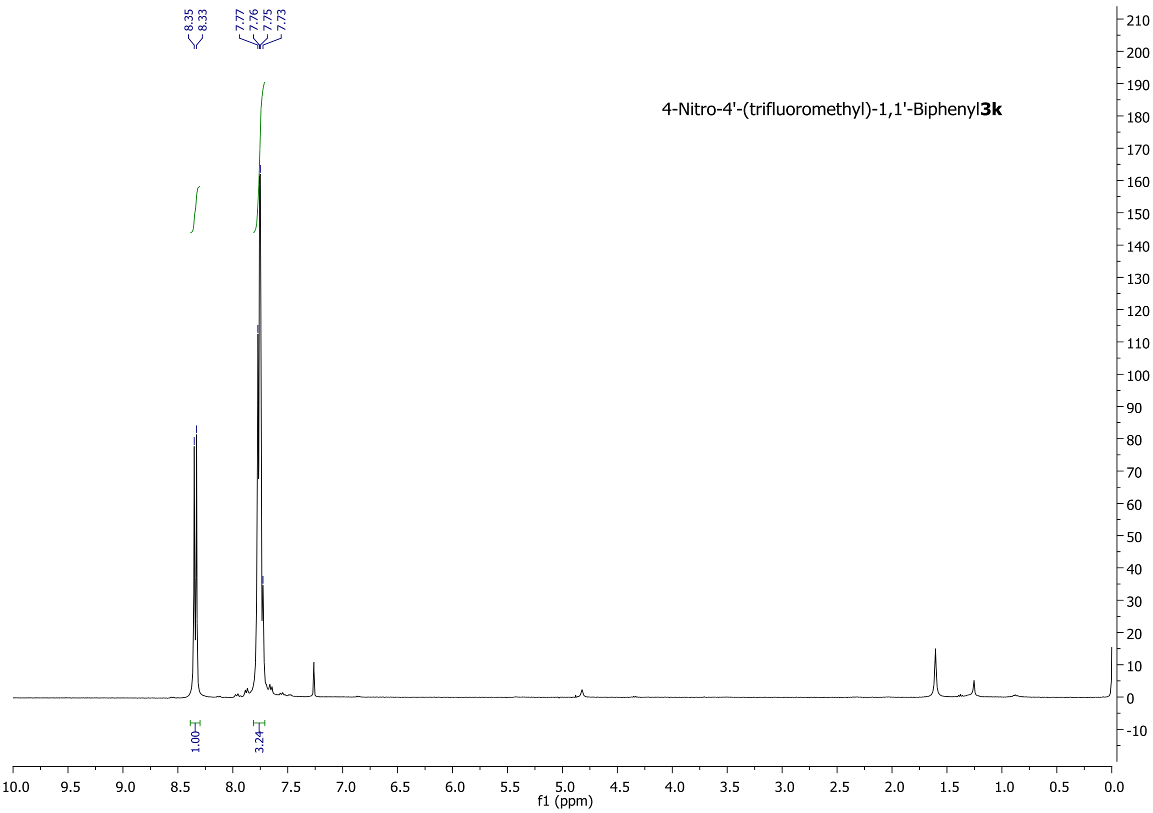

8.35
8.33
7.77
7.76
7.75
7.73
4-Nitro-4'-(trifluoromethyl)-1,1'-Biphenyl**3k**
1.00
3.24
f1 (ppm)

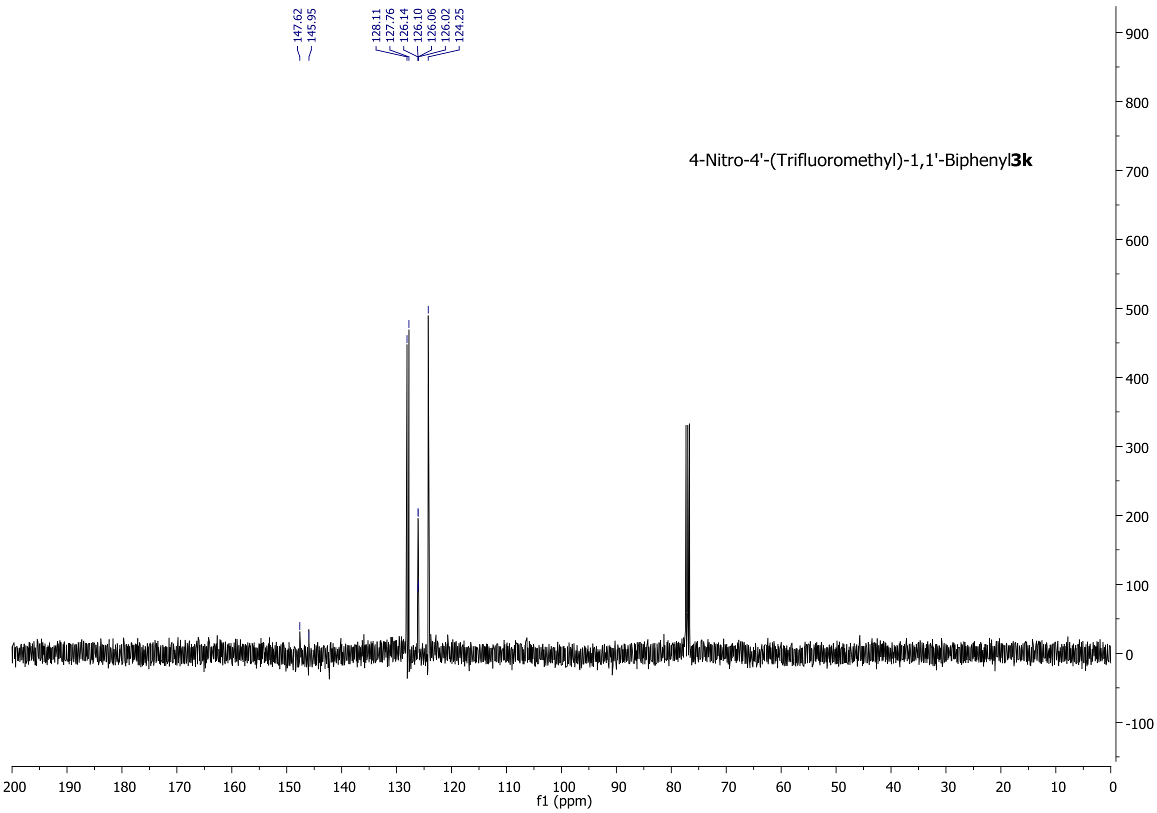

147.62
145.95
128.11
127.76
126.14
126.10
126.06
126.02
124.25
4-Nitro-4'-(Trifluoromethyl)-1,1'-Biphenyl**3k**
f1 (ppm)

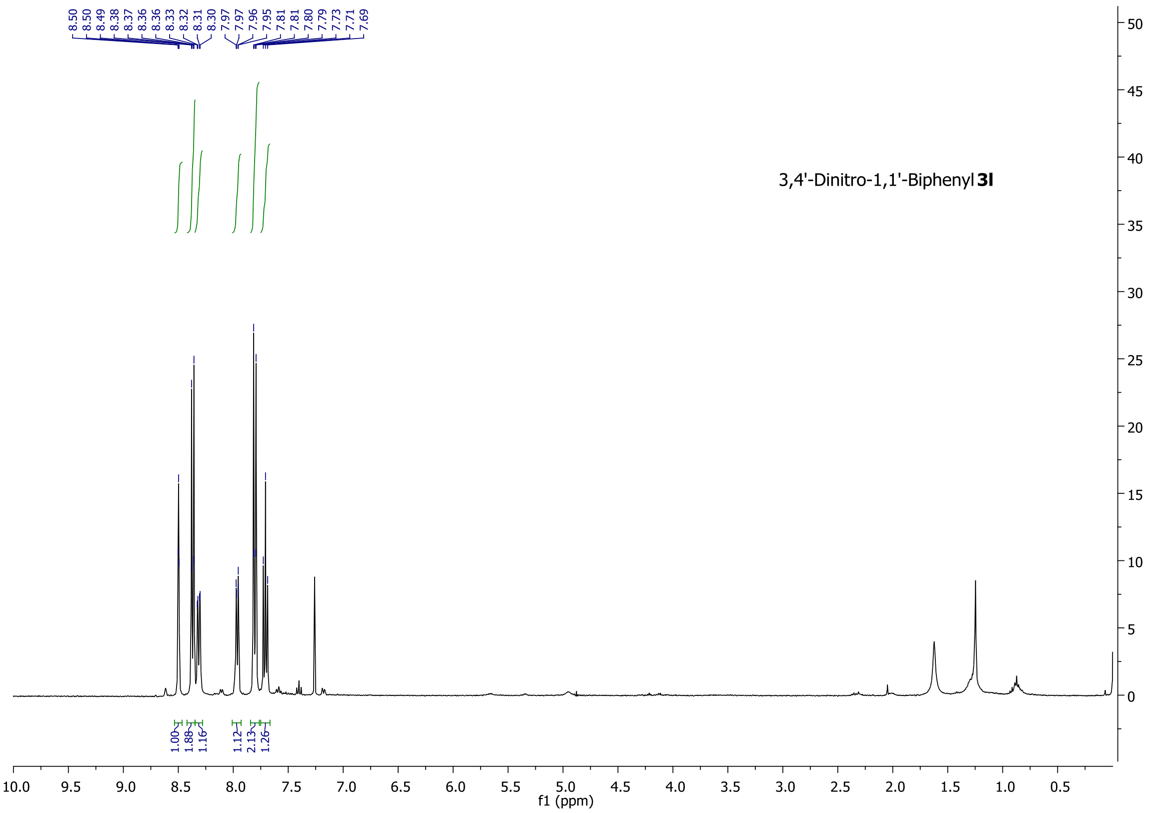
8.50
8.50
8.49
8.38
8.37
8.36
8.36
8.33
8.32
8.31
8.30
7.97
7.97
7.96
7.95
7.81
7.81
7.80
7.79
7.73
7.71
7.69
1.00
1.88
1.16
1.12
2.13
1.26
3,4'-Dinitro-1,1'-Biphenyl 3l
f1 (ppm)

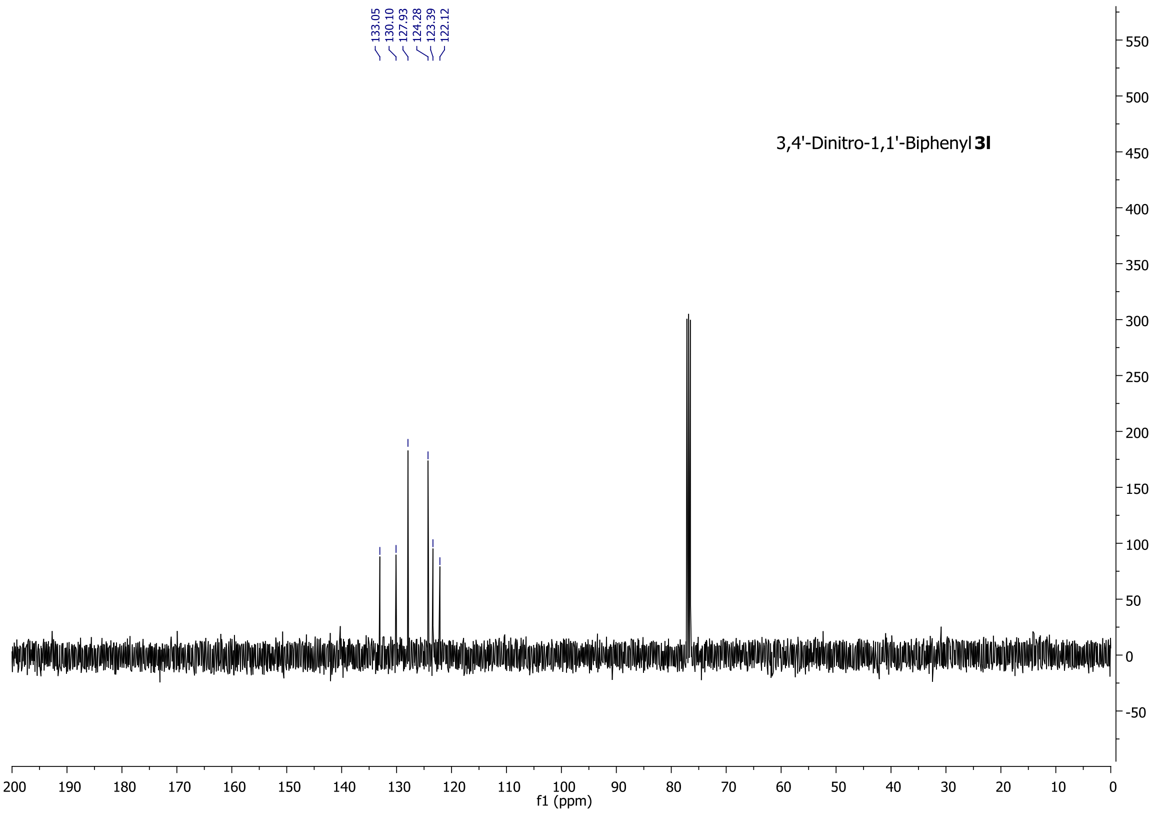
133.05
130.10
127.93
124.28
123.39
122.12
3,4'-Dinitro-1,1'-Biphenyl 3l
f1 (ppm)

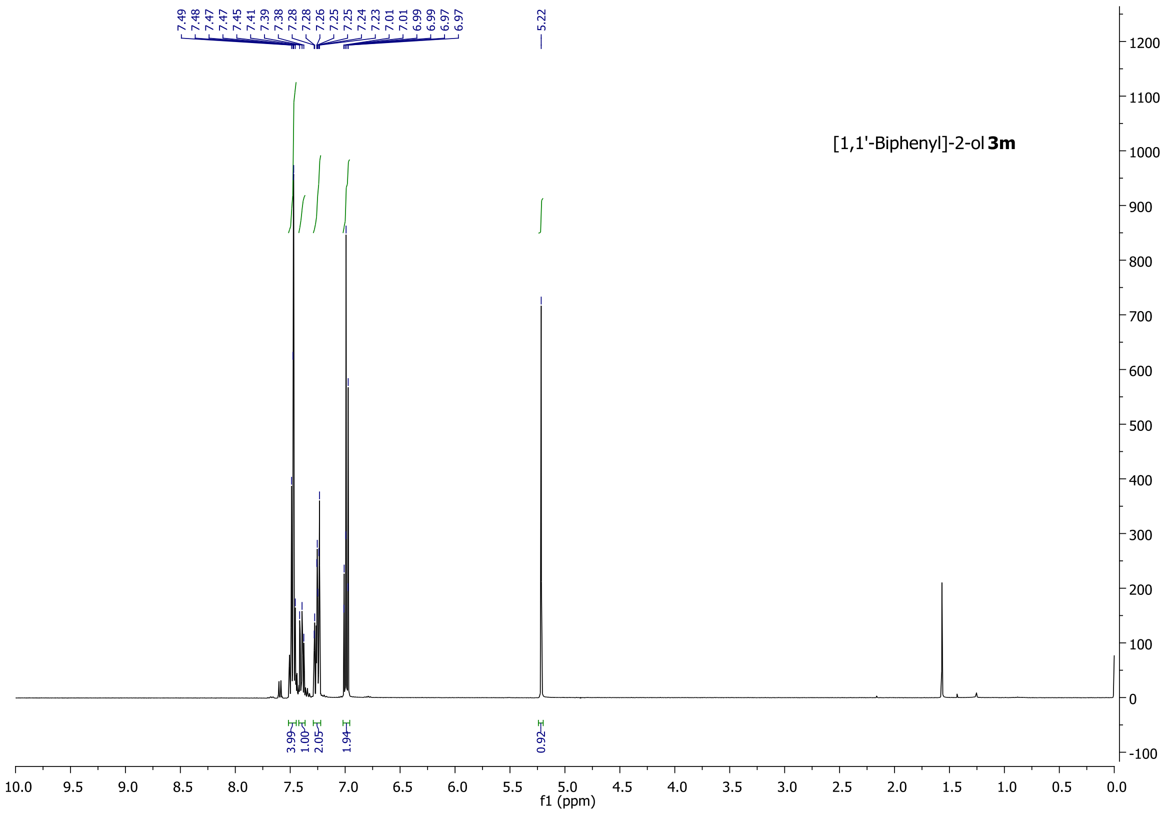
[1,1'-Biphenyl]-2-ol **3m**
f1 (ppm)

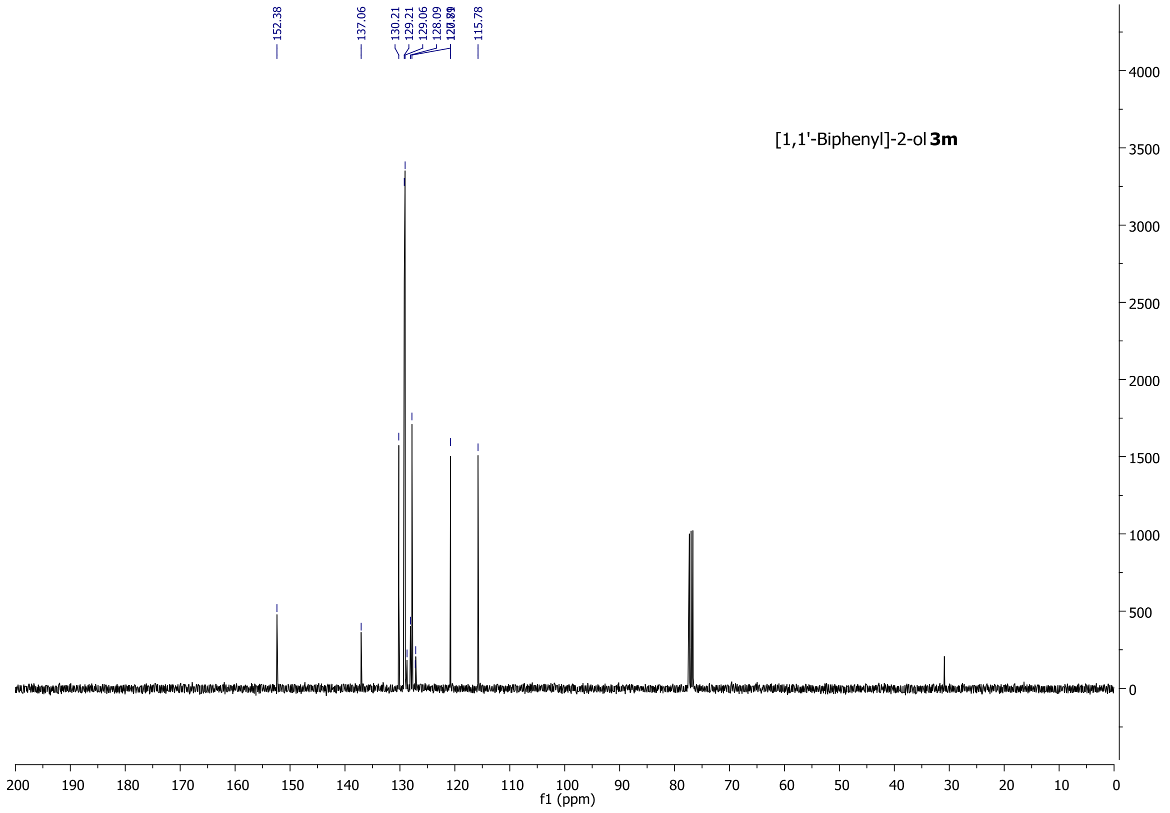
[1,1'-Biphenyl]-2-ol **3m**
152.38
137.06
130.21
129.21
129.06
128.09
115.78
f1 (ppm)

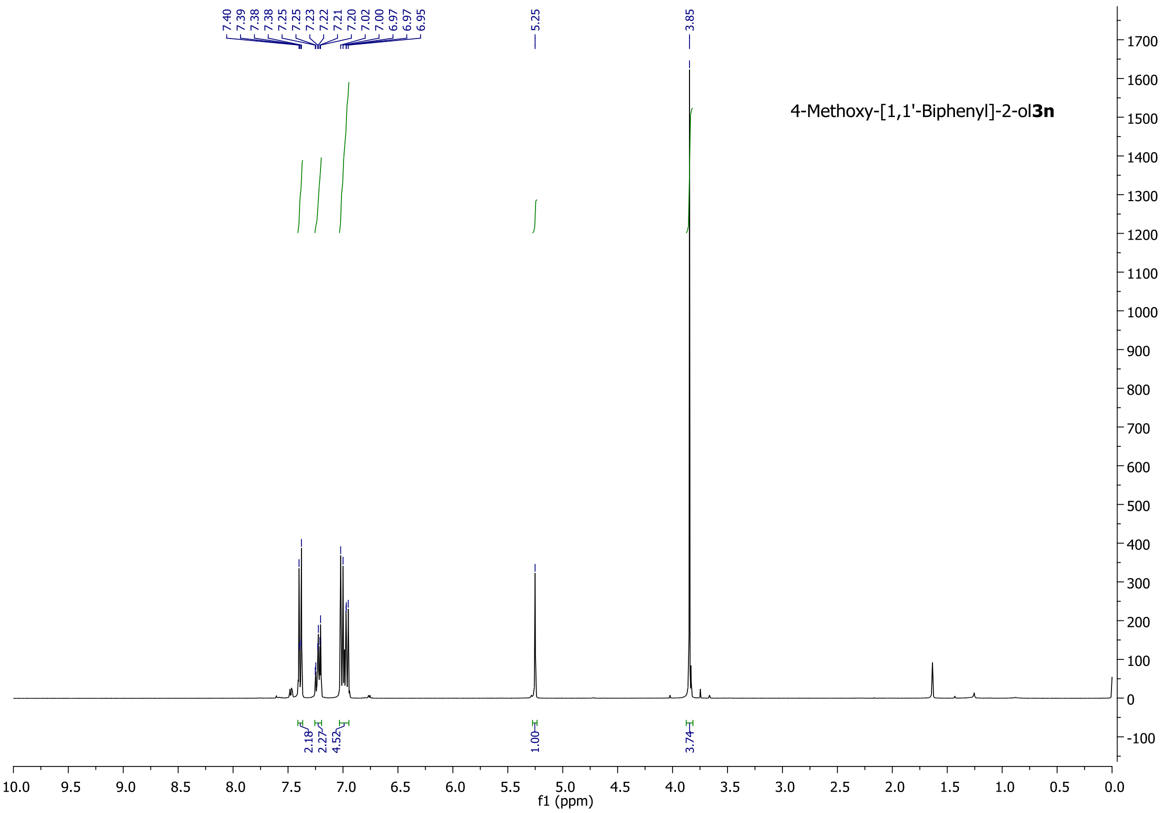
4-Methoxy-[1,1'-Biphenyl]-2-ol3n
f1 (ppm)

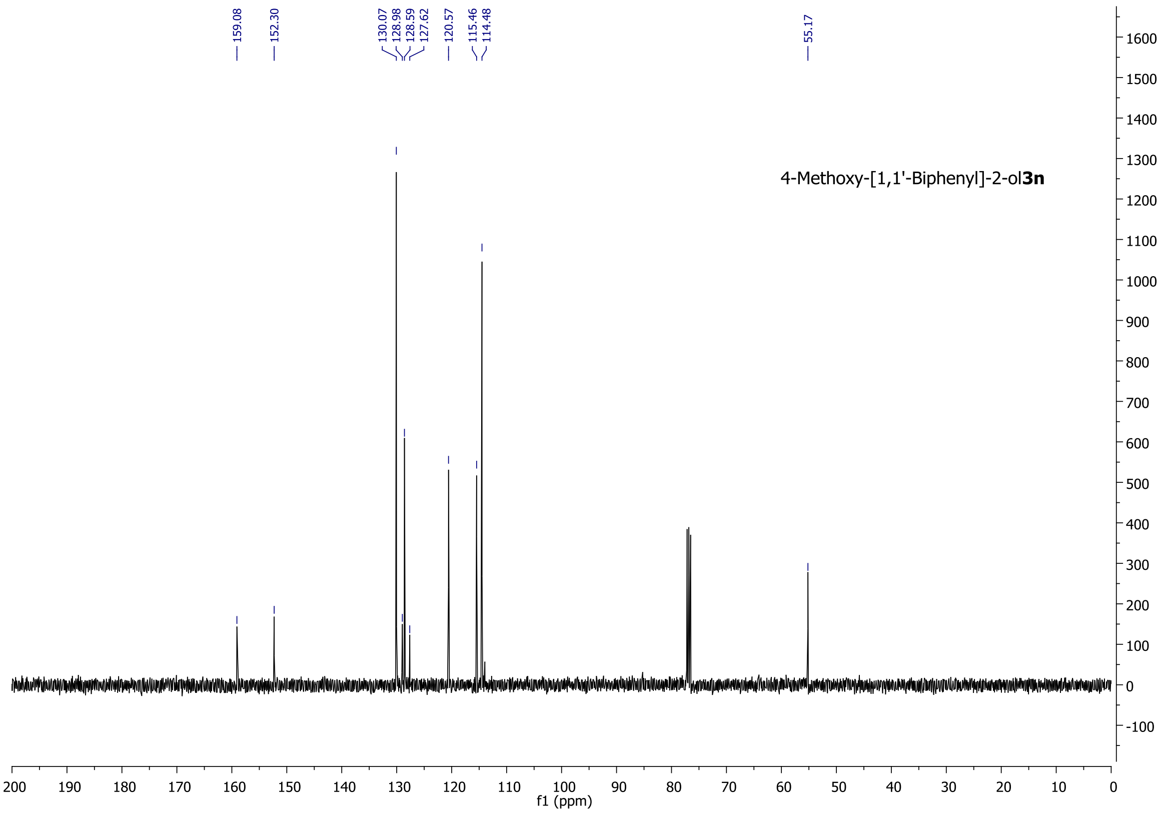
4-Methoxy-[1,1'-Biphenyl]-2-ol3n
f1 (ppm)

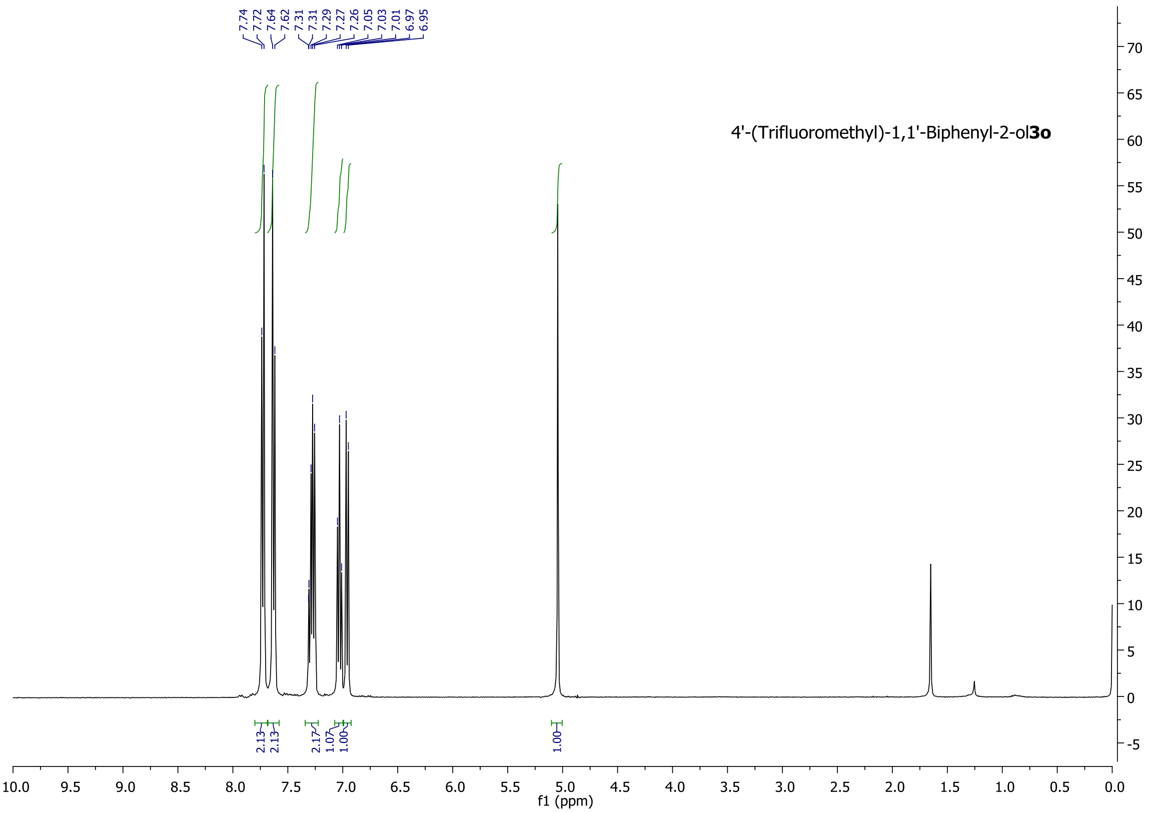
7.74
7.72
7.64
7.62
7.31
7.31
7.29
7.27
7.26
7.05
7.03
7.01
6.97
6.95
4'-(Trifluoromethyl)-1,1'-Biphenyl-2-ol**3o**
2.13
2.13
2.17
1.07
1.00
1.00
f1 (ppm)

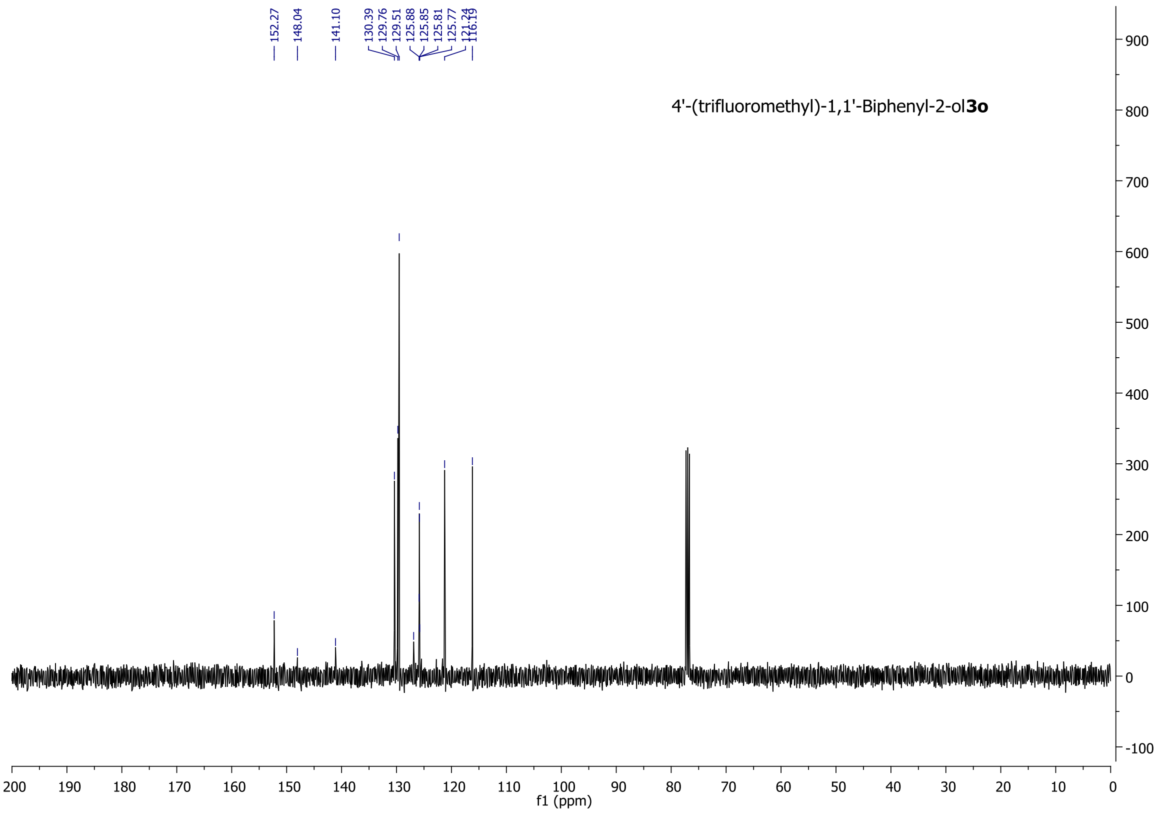
152.27
148.04
141.10
130.39
129.76
129.51
125.88
125.85
125.81
125.77
121.24
116.19
4'-(trifluoromethyl)-1,1'-Biphenyl-2-ol**3o**
f1 (ppm)

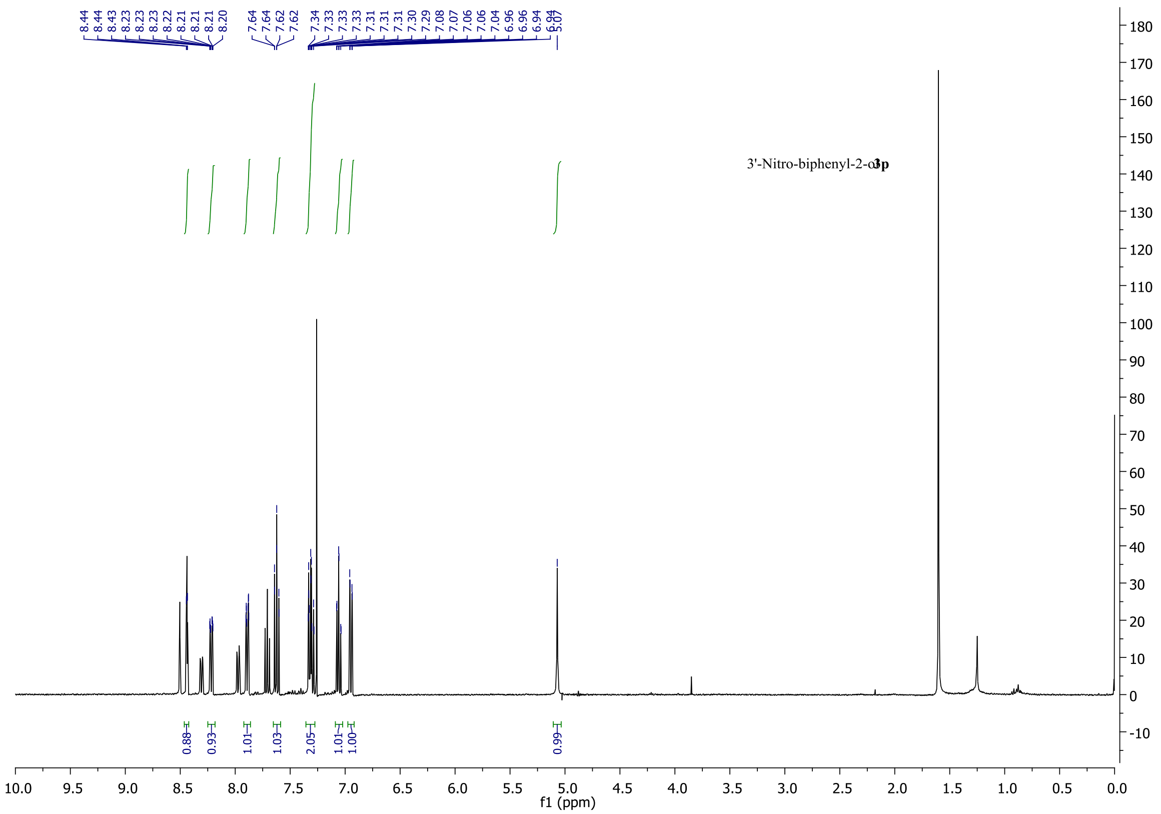

3'-Nitro-biphenyl-2-ol 3p
f1 (ppm)

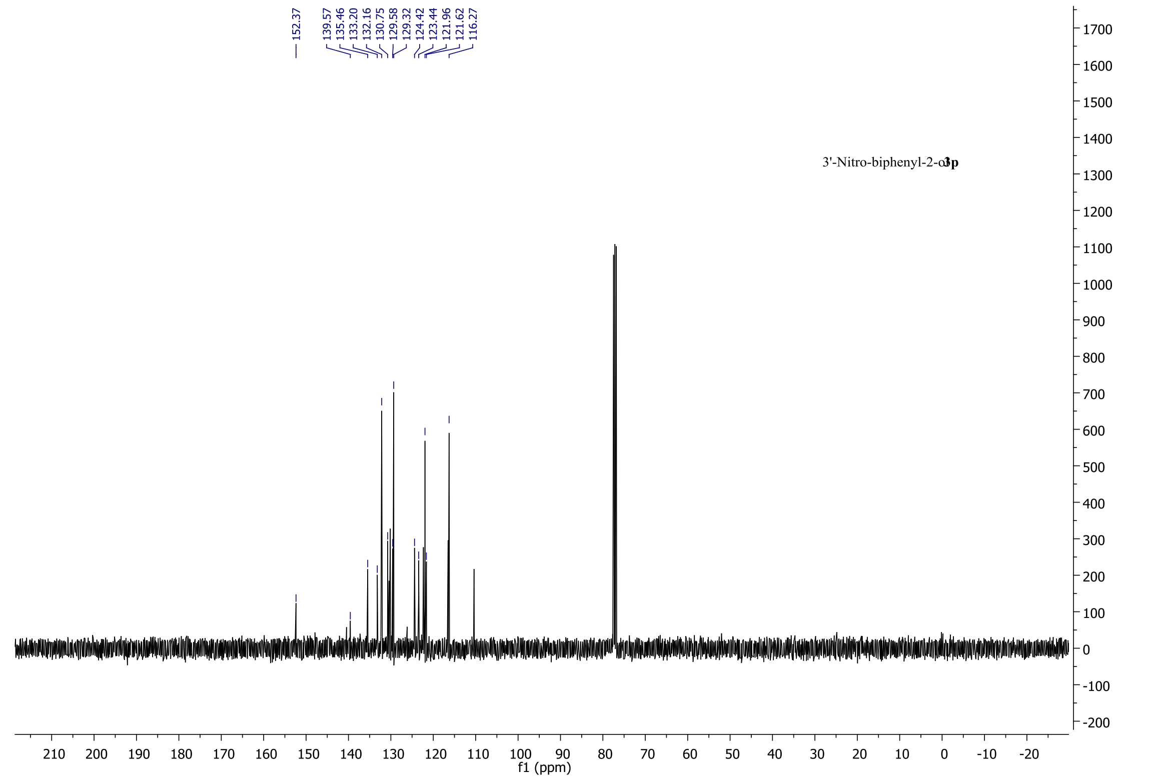

3'-Nitro-biphenyl-2-ol 3p
f1 (ppm)

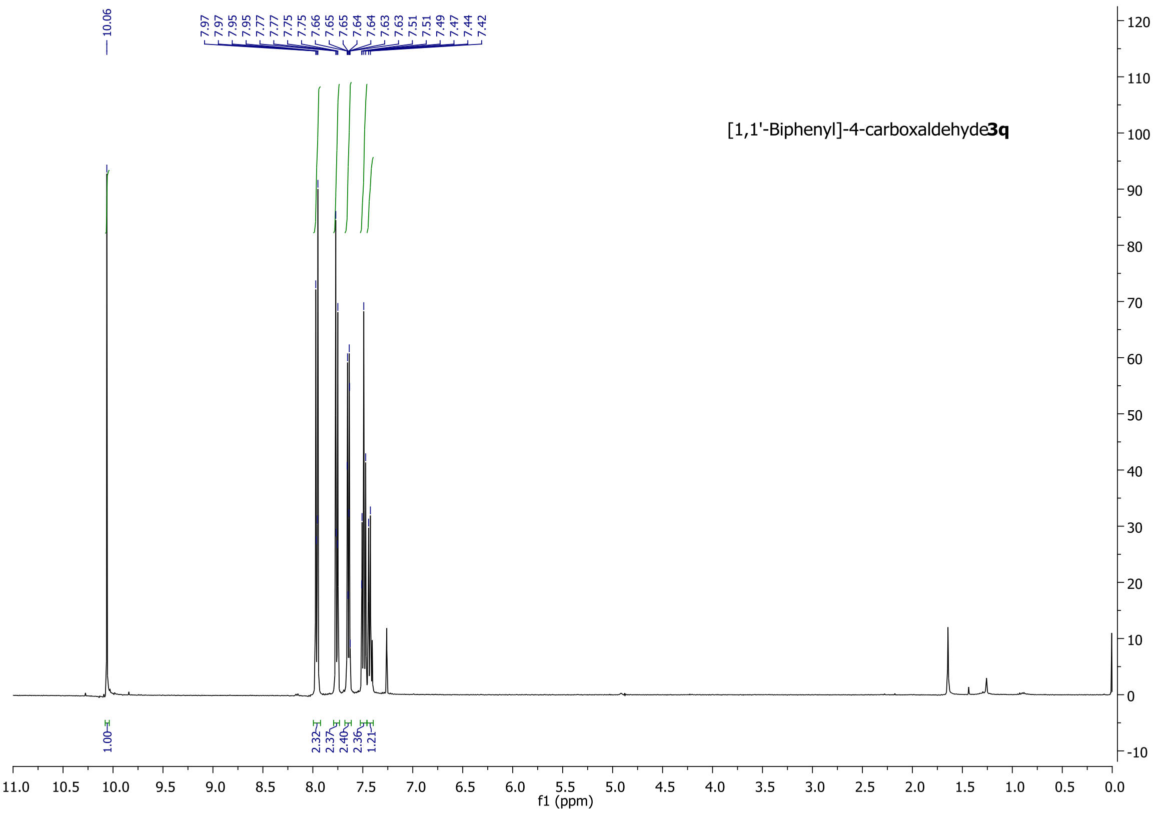
[1,1'-Biphenyl]-4-carboxaldehyde3q
f1 (ppm)

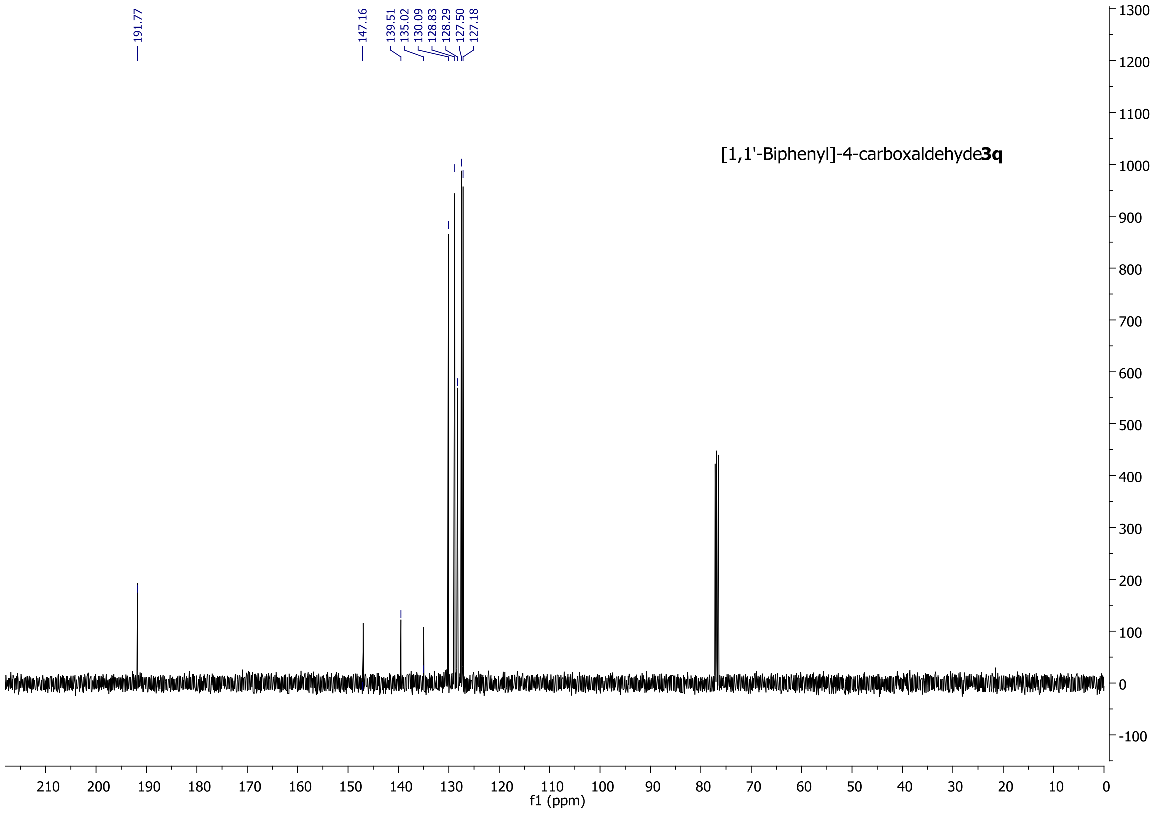
[1,1'-Biphenyl]-4-carboxaldehyde3q
f1 (ppm)

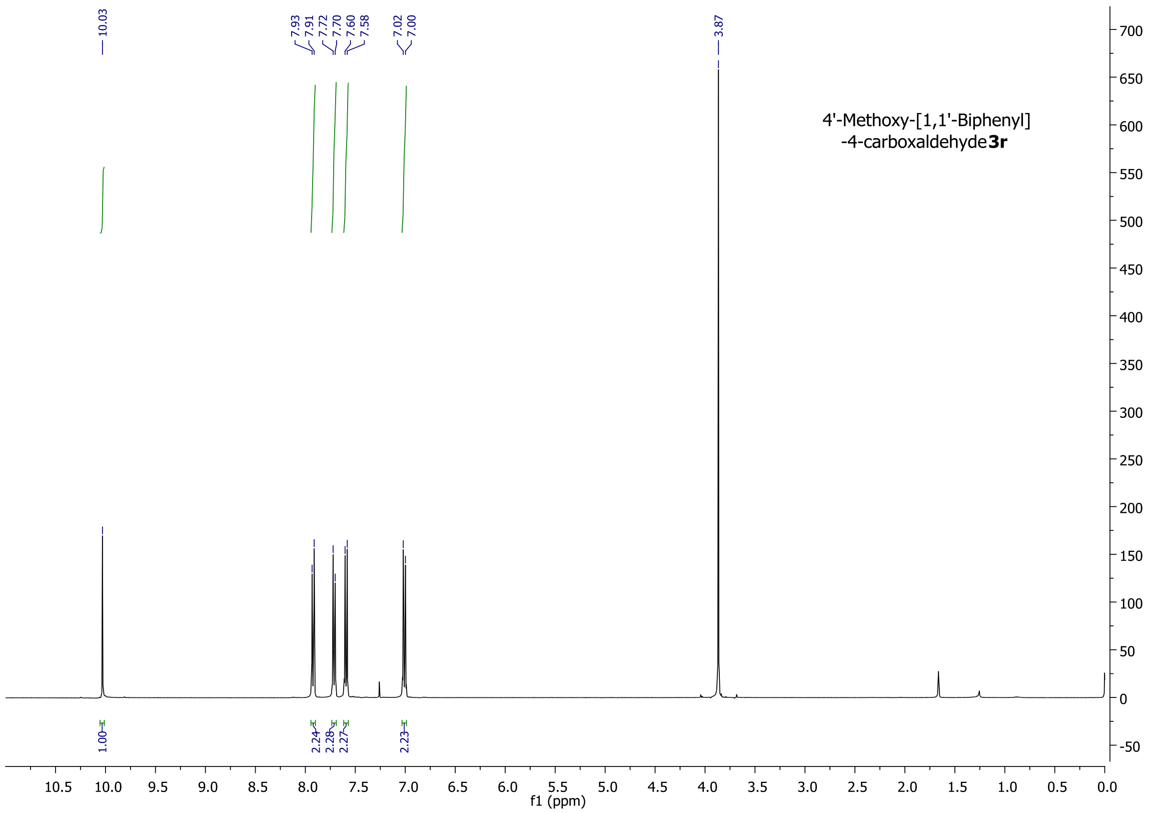
10.03
7.93
7.91
7.72
7.70
7.60
7.58
7.02
7.00
3.87
4'-Methoxy-[1,1'-Biphenyl]
-4-carboxaldehyde**3r**
1.00
2.24
2.28
2.27
2.23
f1 (ppm)

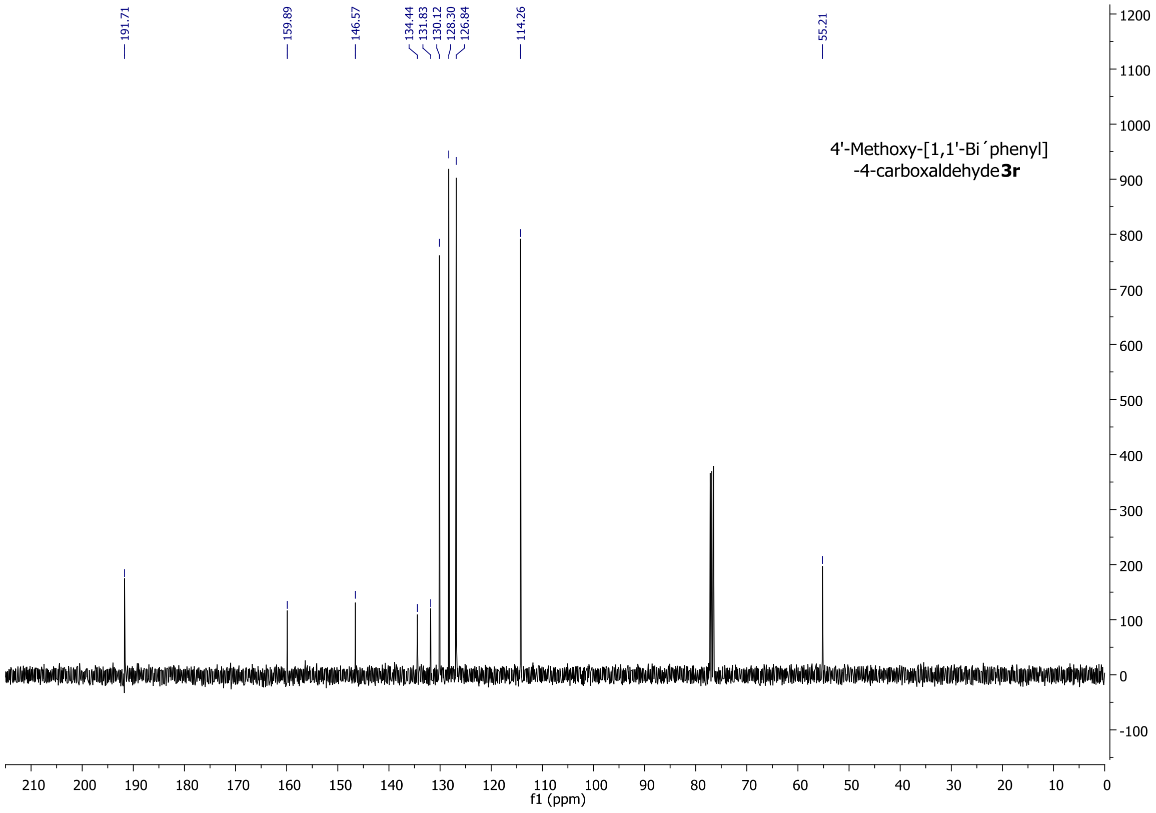
191.71
159.89
146.57
134.44
131.83
130.12
128.30
126.84
114.26
55.21
4'-Methoxy-[1,1'-Bi´phenyl]
-4-carboxaldehyde**3r**
f1 (ppm)

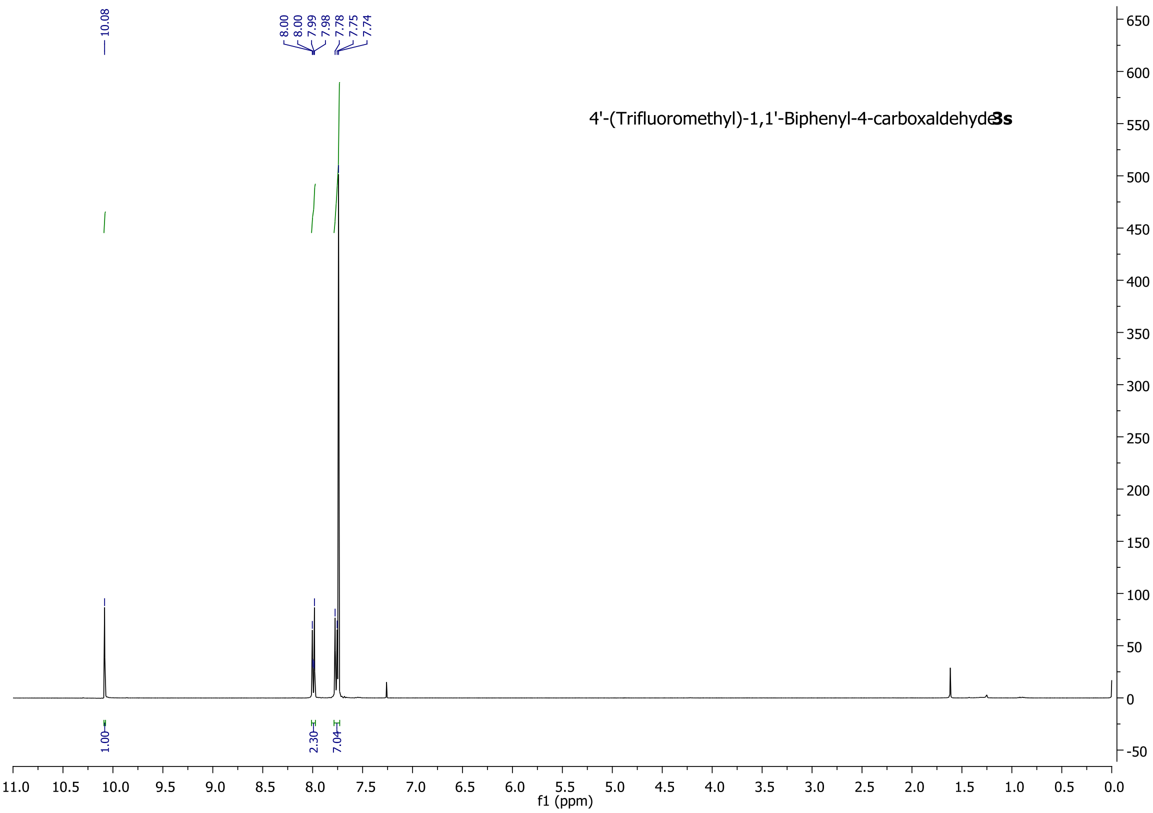
4'-(Trifluoromethyl)-1,1'-Biphenyl-4-carboxaldehyde3s
10.08
8.00
8.00
7.99
7.98
7.78
7.75
7.74
1.00
2.30
7.04
f1 (ppm)

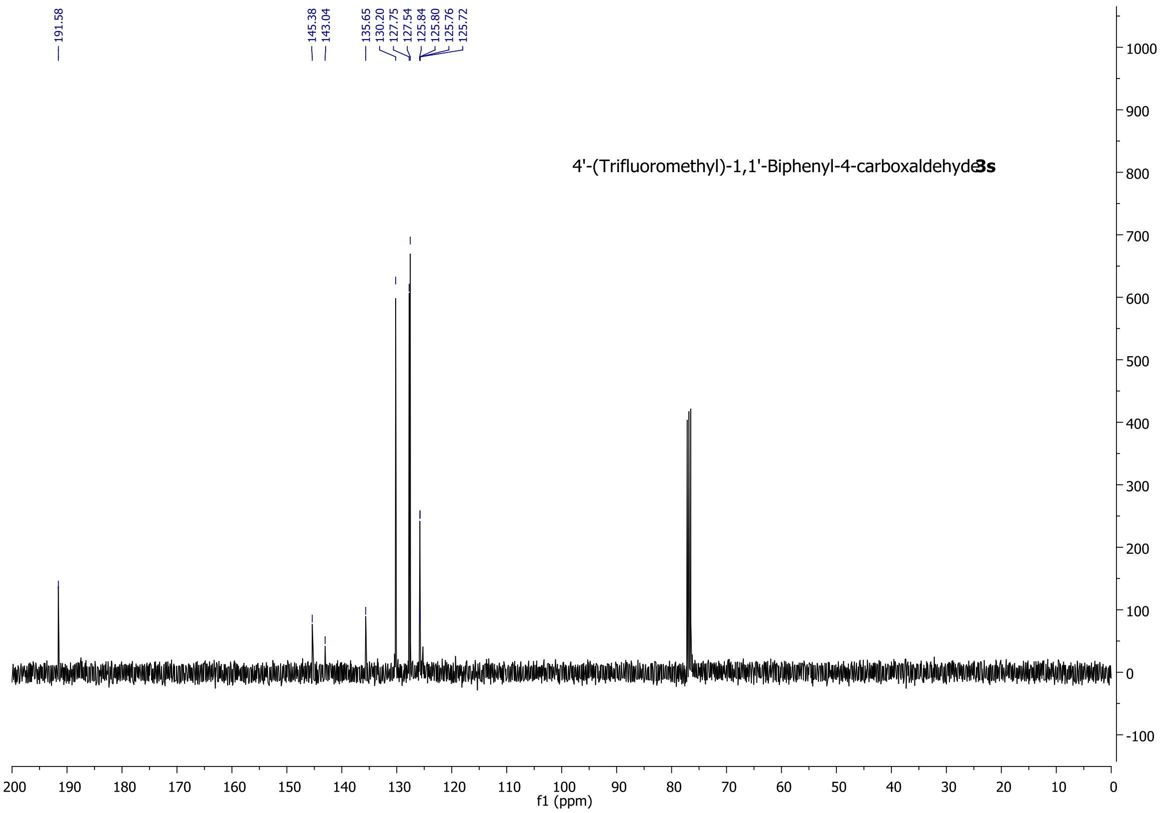
4'-(Trifluoromethyl)-1,1'-Biphenyl-4-carboxaldehyde3s
191.58
145.38
143.04
135.65
130.20
127.75
127.54
125.84
125.80
125.76
125.72
f1 (ppm)

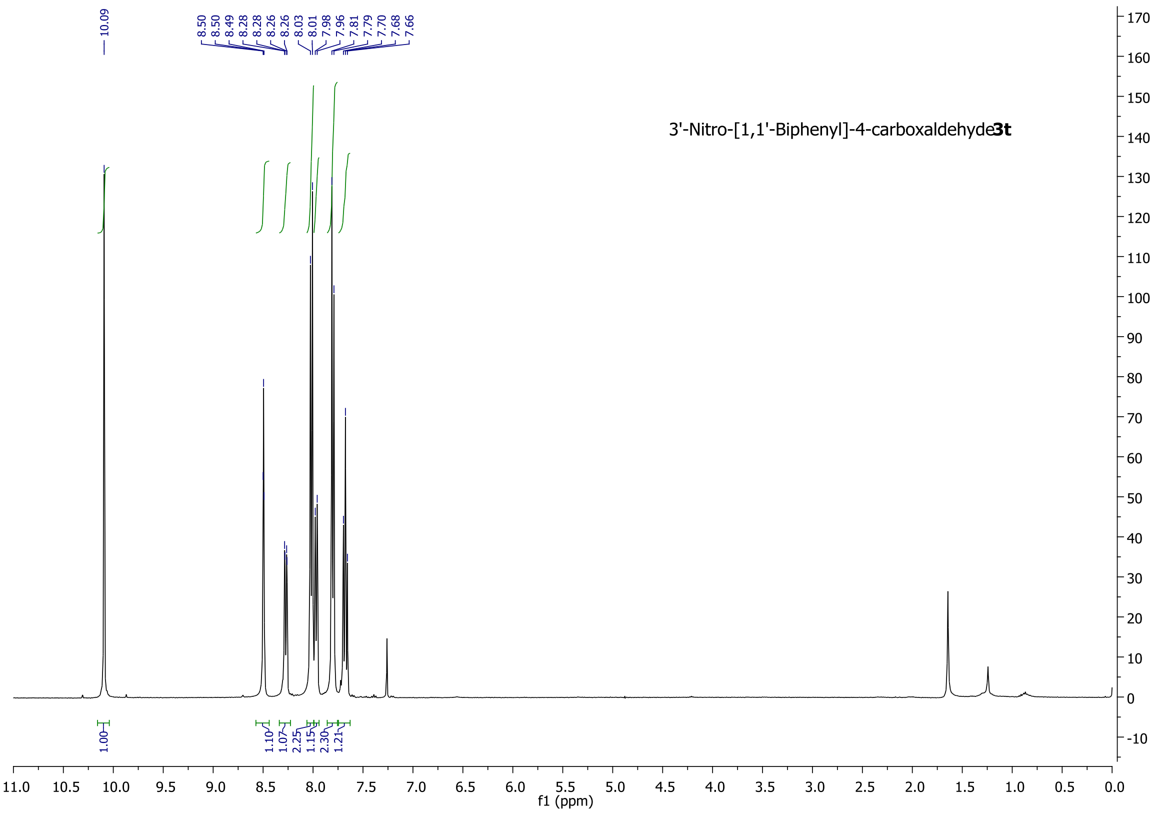
3'-Nitro-[1,1'-Biphenyl]-4-carboxaldehyde 3t
10.09
8.50 8.50 8.49 8.28 8.28 8.26 8.26 8.03 8.01 7.98 7.96 7.81 7.79 7.70 7.68 7.66
1.00 1.10 1.07 2.25 1.15 2.30 1.21
f1 (ppm)

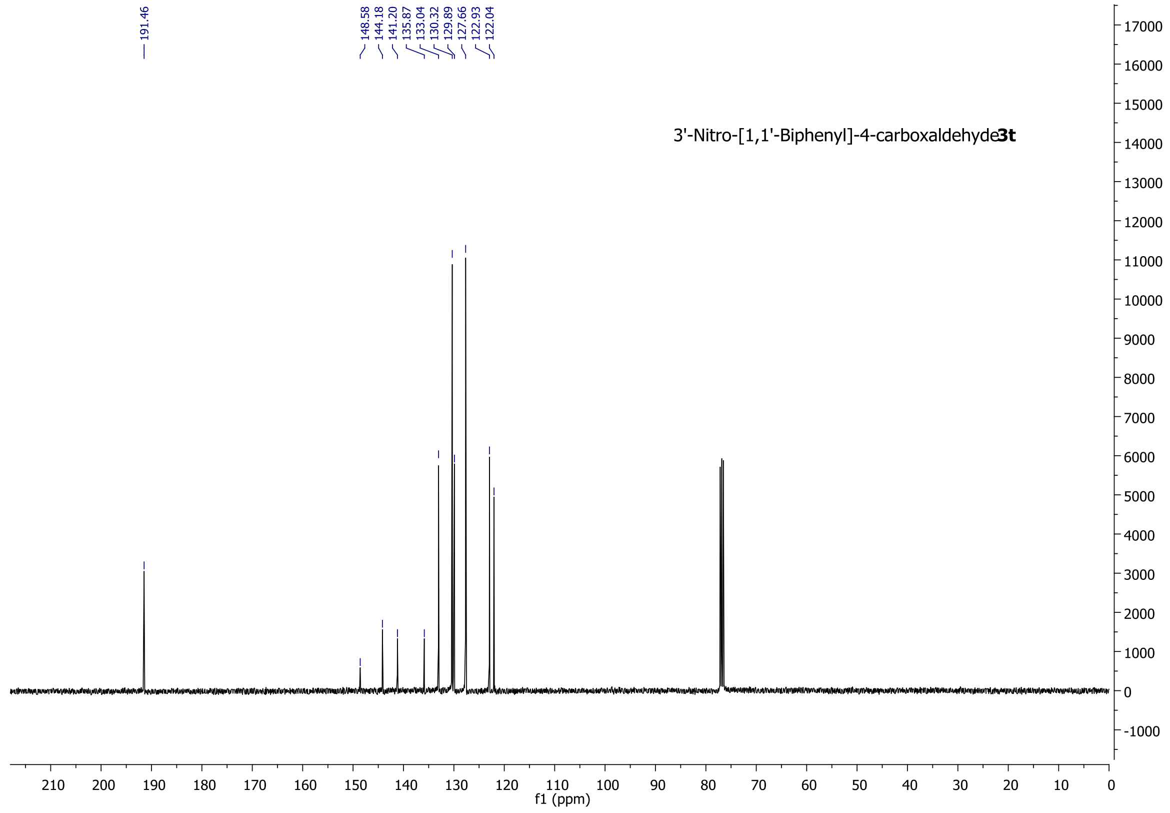
3'-Nitro-[1,1'-Biphenyl]-4-carboxaldehyde 3t
191.46
148.58 144.18 141.20 135.87 133.04 130.32 129.89 127.66 122.93 122.04
f1 (ppm)

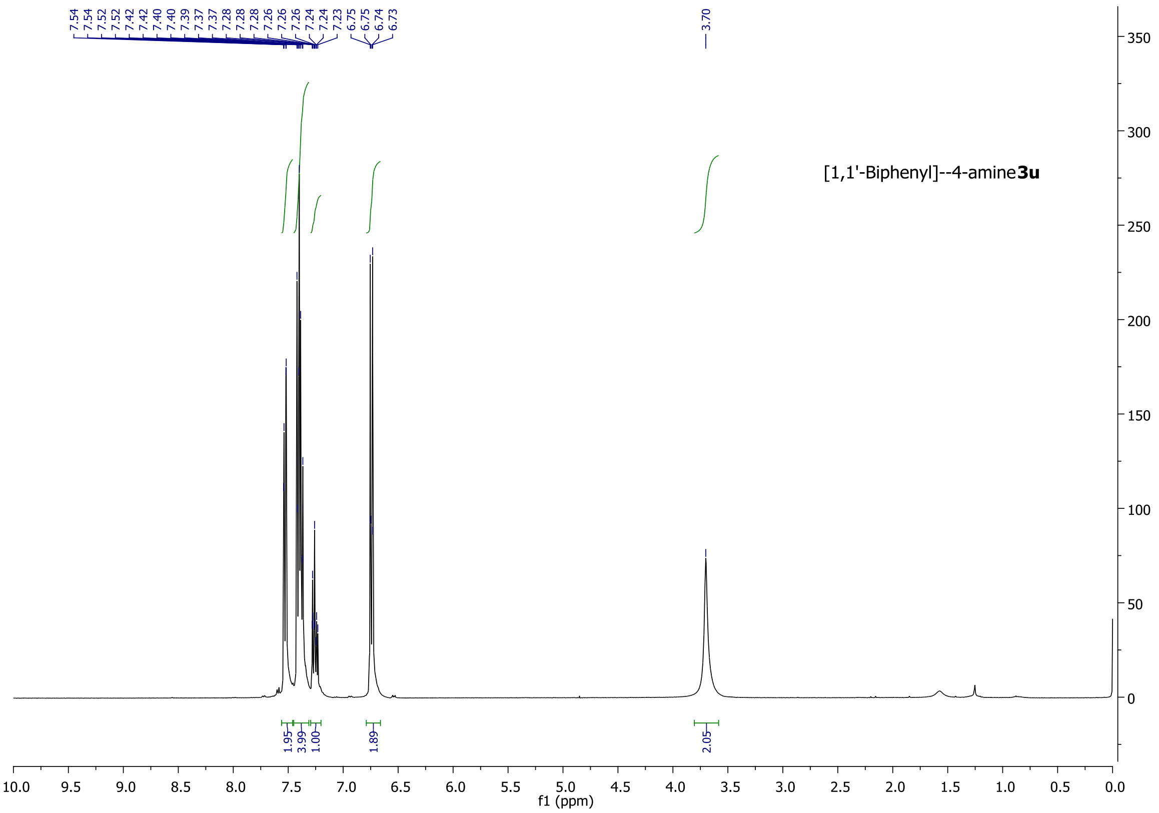

7.54
7.54
7.52
7.52
7.42
7.42
7.40
7.40
7.39
7.37
7.37
7.28
7.28
7.28
7.26
7.26
7.26
7.24
7.24
7.23
6.75
6.75
6.74
6.73
3.70
[1,1'-Biphenyl]--4-amine3u
1.95
3.99
1.00
1.89
2.05
f1 (ppm)

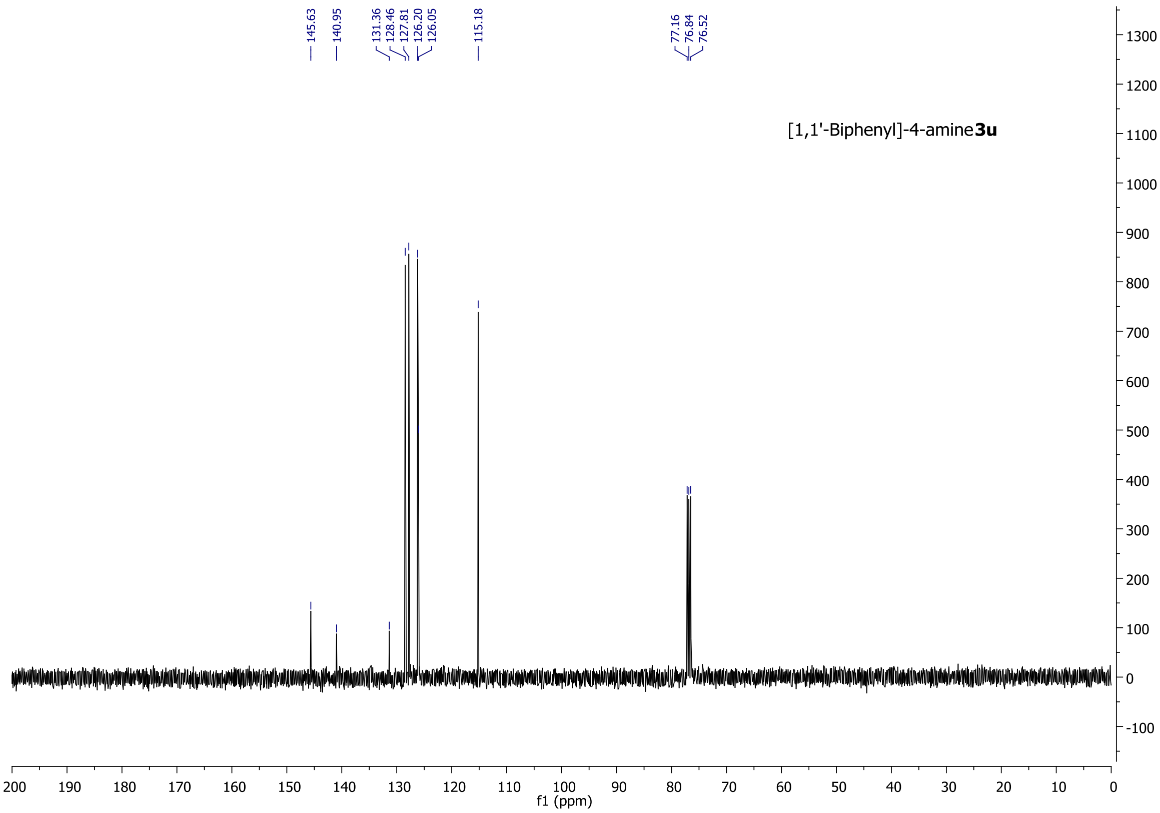

145.63
140.95
131.36
128.46
127.81
126.20
126.05
115.18
77.16
76.84
76.52
[1,1'-Biphenyl]-4-amine3u
f1 (ppm)

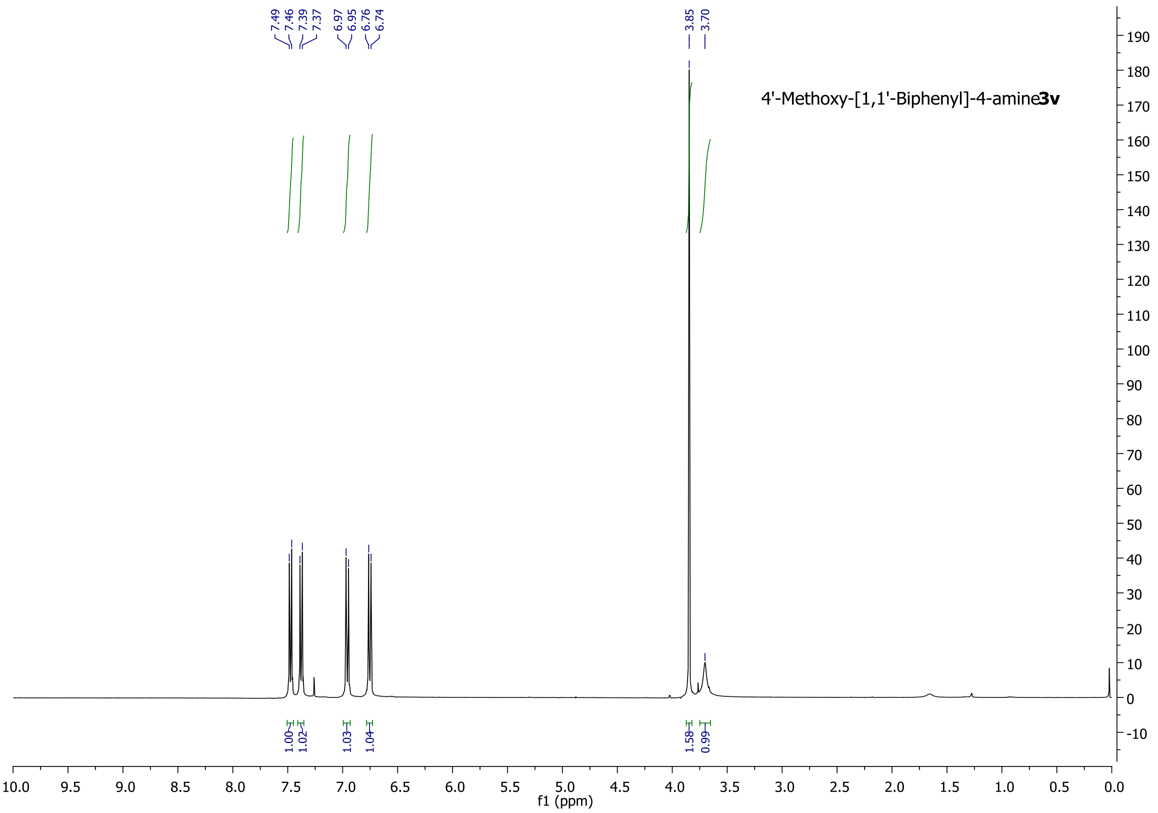

7.49
7.46
7.39
7.37
6.97
6.95
6.76
6.74
3.85
3.70
4'-Methoxy-[1,1'-Biphenyl]-4-amine**3v**
1.00
1.02
1.03
1.04
1.58
0.99
f1 (ppm)

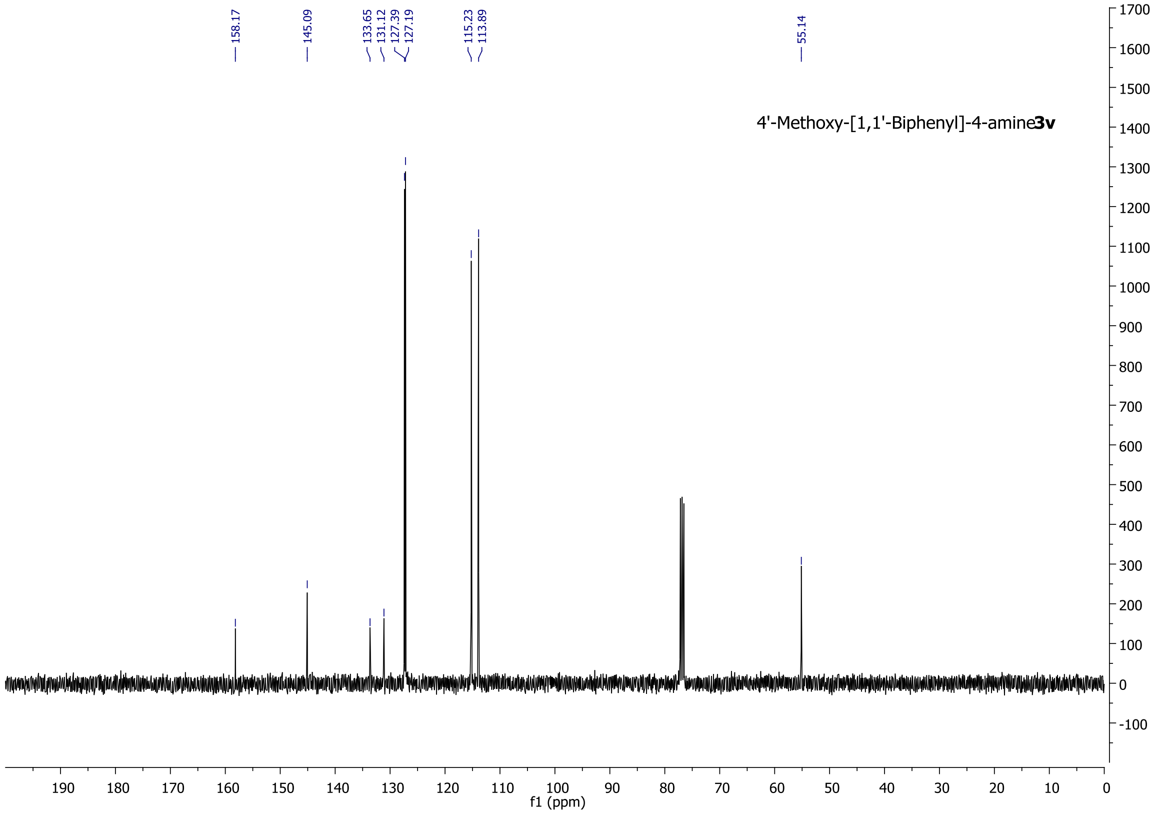

158.17
145.09
133.65
131.12
127.39
127.19
115.23
113.89
55.14
4'-Methoxy-[1,1'-Biphenyl]-4-amine**3v**
f1 (ppm)

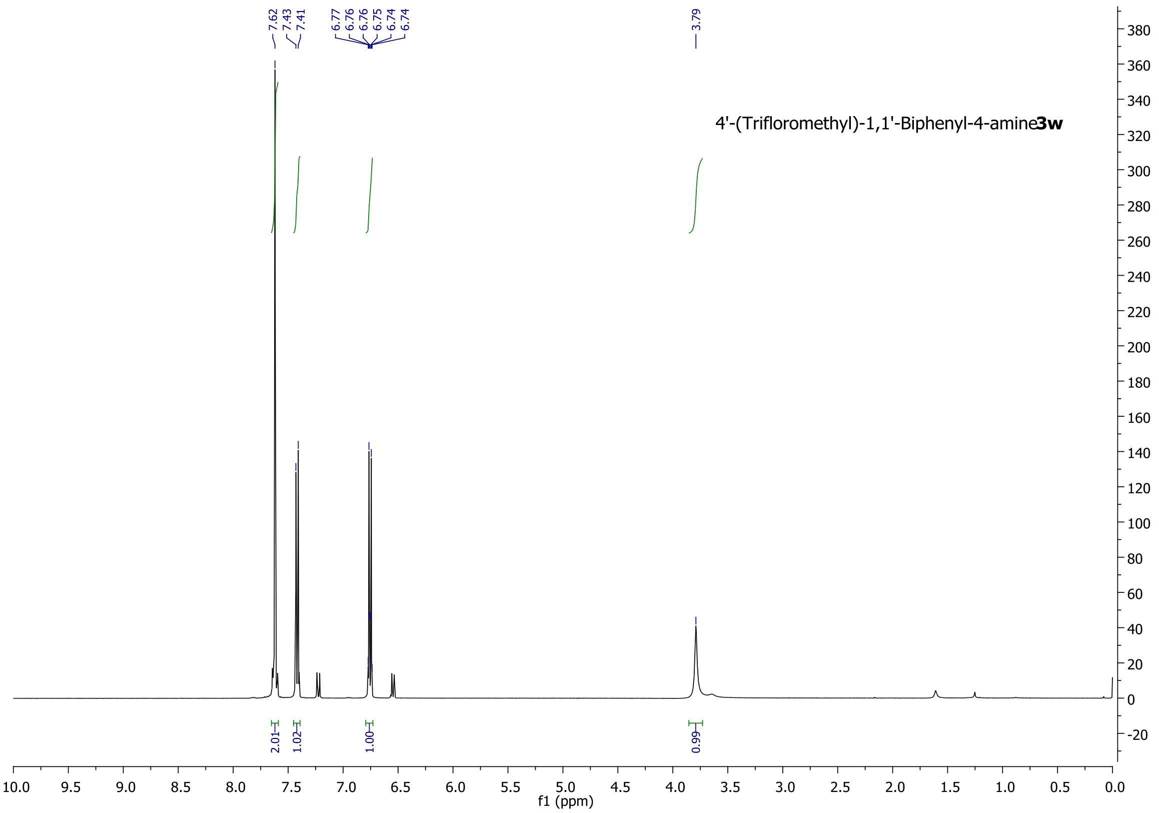
4'-(Trifloromethyl)-1,1'-Biphenyl-4-amine**3w**
7.62
7.43
7.41
6.77
6.76
6.76
6.75
6.74
6.74
3.79
2.01
1.02
1.00
0.99
f1 (ppm)

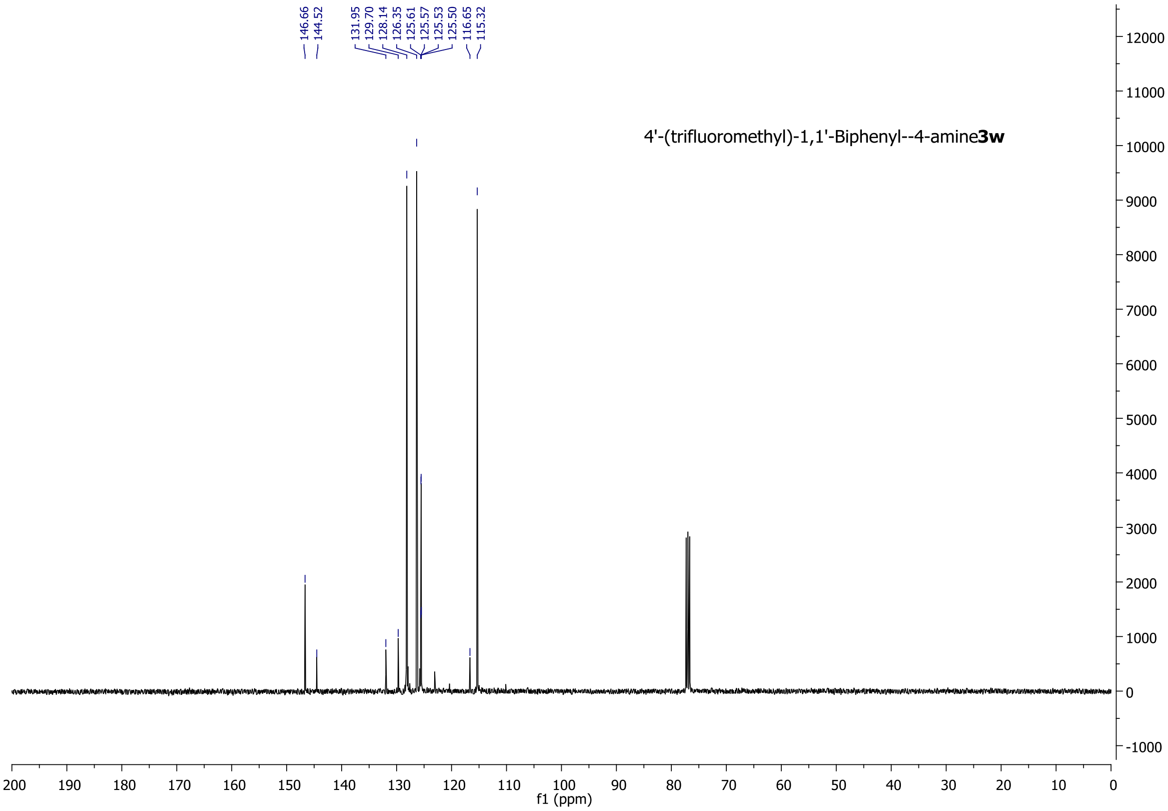
4'-(trifluoromethyl)-1,1'-Biphenyl--4-amine**3w**
146.66
144.52
131.95
129.70
128.14
126.35
125.61
125.57
125.53
125.50
116.65
115.32
f1 (ppm)

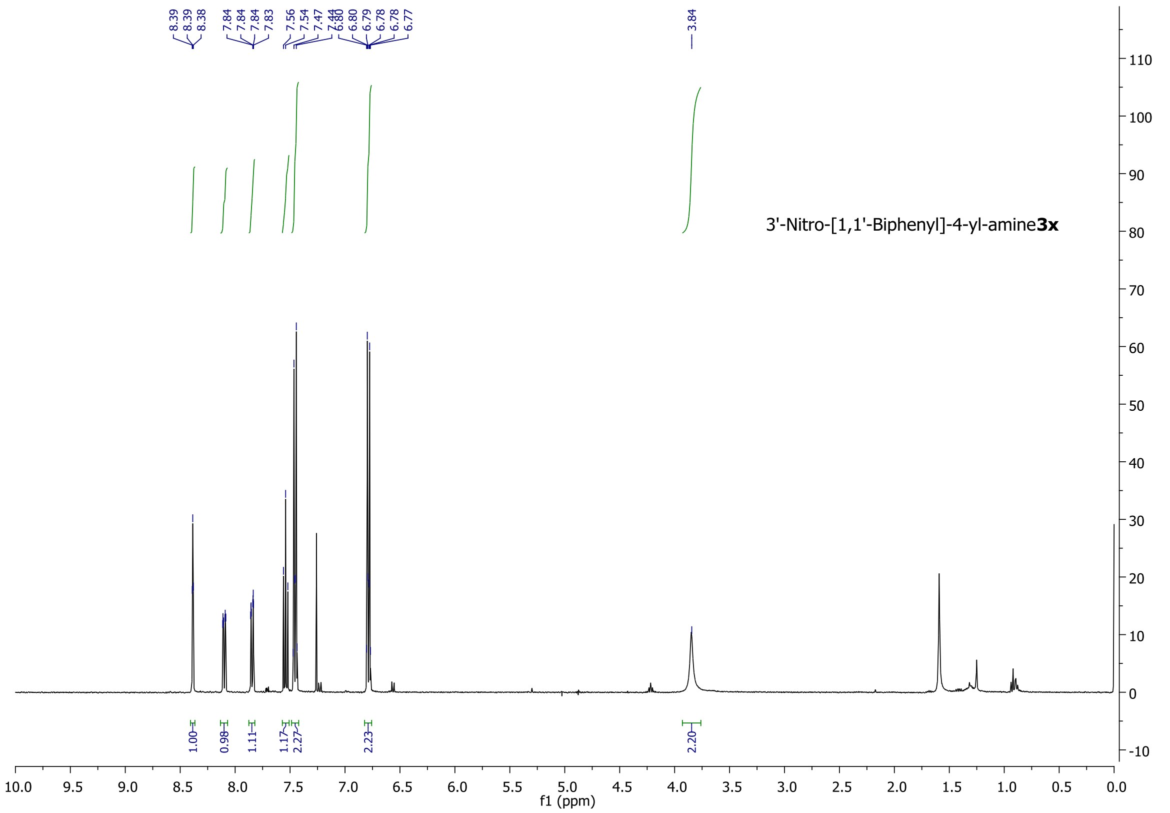
3'-Nitro-[1,1'-Biphenyl]-4-yl-amine**3x**
f1 (ppm)

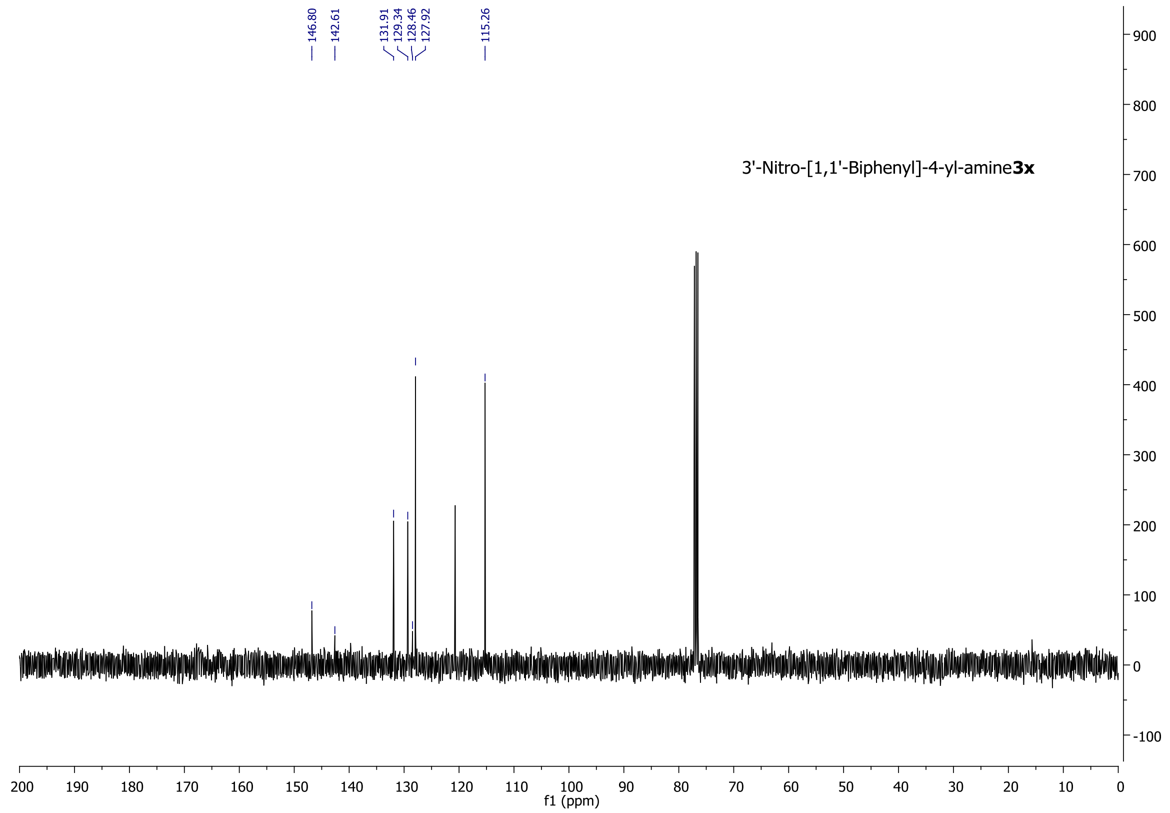
3'-Nitro-[1,1'-Biphenyl]-4-yl-amine**3x**
146.80
142.61
131.91
129.34
128.46
127.92
115.26
f1 (ppm)

References:

[1] Onoue, H.; Minami, K.; Nakagawa, K. *Bull. Chem. Soc. Jpn.* **1970**, *43*, 3480-3485.

[2] Z. G. Zhou, J. C. Shi, Q. S. Hu, Y. R. Xie, Z. Y. Du, S. Y. Zhang, *Appl. Organomet. Chem.* **2011**, *25*, 616.

[3] Alacid, E.; Nájera, C. *Org. Lett.*, **2008**, *10*, 5011

[4] Ackermann, L.; Potukuchi, H. K.; Althammer, A.; Born, R.; Mayer, P. *Org. Lett*., **2010**, *12*, 1004.

[5] Feiran Chen, F.; Huang, M.; Li, Y. *Ind. Eng. Chem. Res.*, **2014**, *53 (20)*, 8339–8345.

[6] Mejías, N.; Pleixats, R.; Shafir, A.; Medio-Simón, M.; Asensio, G. *Eur. J. Org. Chem*., **2010**, 5090–5099.

[7] Ackermann, L.; Kapdi, A. R.; Fenner, S.; Kornhaaß, C.; Schulzke, C. *Chem. Eur. J.*, **2011**, *17*, 2965–2971.

[8] Hwang, S. J.; Kim, H. J.; Chang*, S. Org. Lett.*, **2009**, *11 (20)*, 4588–4591.

[9] Deraedt, C.; Salmon, L.; Ruiz, J.; Astruc, D. *Adv. Synth. Catal.*, **2013**, *355*, 2992–3001.

[10] Liu, L.; Dong, Y.; Pang, B.; Ma, J. *J. Org. Chem.*, **2014**, *79 (15),* 7193–7198.

[11] Zhou, L.; Lu, W. *Organometallics*, **2012**, *31 (6)*, 2124–2127.

[12] Q. Yang, S.M. Ma, J.X. Li, F.S. Xiao, H. Xiong, *Chem. Commun.,* **2006**, 2495.

[13] Inamoto, K.; Kadokawa, J.; Kondo, Y. *Org. Lett.*, **2013**, *15 (15)*, 3962–3965.

[14] Ren, H.; Xu, Y.; Jeanneau, E.; Bonnamout, I.; Tu, T.; Darbost, U. *Tetrahedron*, **2014**, *70*, 2829-2837.

[15] Hirner, J. J.; Blum, S. A. *Organometallics*, **2011**, *30 (6)*, 1299–1302.

[16] Alizadeh, A.; Khodaei, M. M.; Kordestani, D.; Beygzadeh, M. *Tetrahedron Lett*., **2013**, *54(4)*, 291-294.

[17] Das, S.; Bhunia, S.; Maity, T.; Koner, S. *J. Mol. Catal. A-Chem.*, **2014**, *394*, 188-197.

[18] Peña-López, M.; Sarandeses, L. A.; Pérez Sestelo, J. *Eur. J. Org. Chem*. **2013**, *13*, 2545-2554.
